# Au@Ag Core@Shell Nanoparticles Synthesized with *Rumex hymenosepalus* as Antimicrobial Agent

**DOI:** 10.1186/s11671-021-03572-5

**Published:** 2021-07-22

**Authors:** Jesús Mauro Adolfo Villalobos-Noriega, Ericka Rodríguez-León, César Rodríguez-Beas, Eduardo Larios-Rodríguez, Maribel Plascencia-Jatomea, Aarón Martínez-Higuera, Heriberto Acuña-Campa, Alfonso García-Galaz, Roberto Mora-Monroy, Francisco Javier Alvarez-Cirerol, Blanca Esthela Rodríguez-Vázquez, Roberto Carlos Carillo-Torres, Ramón A. Iñiguez-Palomares

**Affiliations:** 1 Nanotechnology Graduate Program, Department of Physics, University of Sonora, Rosales and Transversal, 83000 Hermosillo, Sonora Mexico; 2 Department of Chemical Engineering and Metallurgy, University of Sonora, Rosales and Transversal, 83000 Hermosillo, Sonora Mexico; 3 Department of Research and Postgraduate in Food, University of Sonora, Rosales and Transversal, 83000 Hermosillo, Sonora Mexico; 4 Department of Physics, University of Sonora, Rosales and Transversal, 83000 Hermosillo, Sonora Mexico; 5 Food Science Coordination, Research Center in Food and Development (CIAD), Road Gustavo Enrique Astiazarán Rosas, No. 46, Col. La Victoria, 83304 Hermosillo, Sonora Mexico; 6 Department of Physic Researching, University of Sonora, Rosales and Transversal, 83000 Hermosillo, Sonora Mexico; 7 Health Sciences Graduate Program, Department of Biological Chemies, University of Sonora, Hermosillo, Sonora Mexico; 8 Department of Polymers and Materials Research, University of Sonora, Rosales and Transversal, 83000 Hermosillo, Sonora Mexico

**Keywords:** Au@Ag core@shell nanoparticles, *Rumex hymenosepalus*, Gompertz model, Lag phase

## Abstract

In this work, we used a sequential method of synthesis for gold–silver bimetallic nanoparticles with core@shell structure (Au@AgNPs). *Rumex hymenosepalus* root extract (Rh), which presents high content in catechins and stilbenes, was used as reductor agent in nanoparticles synthesis. Size distribution obtained by Transmission Electron Microscopy (TEM) gives a mean diameter of 36 ± 11 nm for Au@AgNPs, 24 ± 4 nm for gold nanoparticles (AuNPs), and 13 ± 3 nm for silver nanoparticles (AgNPs). The geometrical shapes of NPs were principally quasi-spherical. The thickness of the silver shell over AuNPs is around 6 nm and covered by active biomolecules onto the surface. Nanoparticles characterization included high angle annular dark field images (HAADF) recorded with a scanning transmission electron microscope (STEM), Energy-Dispersive X-ray Spectroscopy (EDS), X-Ray Diffraction (XRD), UV–Vis Spectroscopy, Zeta Potential, and Dynamic Light Scattering (DLS). Fourier Transform Infrared Spectrometer (FTIR), and X-ray Photoelectron Spectroscopy (XPS) show that nanoparticles are stabilized by extract molecules. A growth kinetics study was performed using the Gompertz model for microorganisms exposed to nanomaterials. The results indicate that AgNPs and Au@AgNPs affect the lag phase and growth rate of *Escherichia coli* and *Candida albicans* in a dose-dependent manner, with a better response for Au@AgNPs

**Supplementary Information:**

The online version contains supplementary material available at 10.1186/s11671-021-03572-5.

## Introduction

In the last 25 years, several chemical methods have been studied for nanomaterial synthesis; however, most of these methods use substances that are unfriendly to the environment and use high temperatures or expensive equipment. In this work, we performed the synthesis of gold–silver nanostructures by using the green synthesis method. This method minimizes pollution from the beginning. Using "clean" processes, avoiding most of the waste and use of hazardous pollutants in developing "clean" nanomaterials that do not pose a threat to health or the environment.

Green synthesis of metal nanoparticles seeks a positive influence of the interaction with biological systems, which means that nanoparticles and their self-functionalization with polyphenol molecules generate biological interactions compatible with systems such as cells and macromolecules. In general, these biological interactions are used as nanomedicine for diseases such as cancer, diabetes, and neurodegenerative diseases. The synthesis of silver (shell) and AuNps as a core–shell system has applications such as optical diagnostic sensors, molecular sensor [1, 2], photothermal therapies, antimicrobials and improves the catalytic process [3–6] compared with monometallic NPs.

In particular, sequential or simultaneous methods in different reactors, chemical and physical methods [7] are used for the bimetallic synthesis: ultrasonic spray pyrolysis[8], a sonochemical method[5], a microfluidic chip [9], sequential nanofluidic nanoprecipitation [10, 11] microemulsions[12], liposomes [13], reducing agents used are chemical or green chemical types.

Dry materials are used in nanoparticles synthesis as metal oxides [14], carbon nanotubes [15, 16], other uses printed soft electronic devices [17]. Wet, nanoparticles used on biological systems [18–20] drugs carrier [21, 22], antimicrobial [23, 24], sensing application [25] and Computational nanotechnology are a modern classification [26] used to define uses.

Khatami et al., reviewed the green synthesis using plants of core@shell-type nanoparticles, in particular, were used *Antigonon leptopus*, *Diopyros kaki*, *Azadirachta indica*, *Potamogeton pectinatus*, *Anacadium oceidentale* for the synthesis of Au@Ag nanoparticles with size between 5 and 500 nm (a), were tested for different applications (non-antibacterial), and *Asparagus racemosus* Root Extract was used to synthesis of Au–Ag alloy nanoparticles finding a MIC at 480 µg/mL tested in *Escherichia coli*, *Bacillus subtilis*, *Klebsiella pneumonia*, *Pseudomonas aeruginosa*, and *Staphylococcus aureus* [27]. Au@Ag NPs obtained by chemical synthesis have applications as antibacterial, and the MIC reported is around 2.5 µg/mL for silver content (shell) [28], Lu et. al. reported a chemical synthesis of Au/AgNPs@Van were prepared using NaBH_4_ and addition Vancomycin and MIC was 60 nmol/mL for nanoparticles bimetallic tested in gram-positive and negative bacteria [29].

Antibacterial properties of bimetallic nanomaterials [30–34] improve as a function of the concentration of Ag. In contrast, an increment in Au concentration decreases the antibacterial properties but reduces the cytotoxic effect, i.e., the bimetallic material becomes more biocompatible [35]. Comparing the effect of Au and Ag monometallic materials with bimetallic materials [36–40], it has been demonstrated that a synergistic effect [41] occurs between bimetallic materials, generating bifunctional effects [28, 42].

Nanomaterials functionalization has particular interest since the chemical environment [43–45] (pH, presence of sulfur, biocompatible molecules, etc.) surrounding the system will have effects on the interaction with cells or microorganisms; therefore, the emphasis on generating biomaterials using green chemistry [46–55].

The chemical composition of the bimetallic particles [56–58] will be a determining factor in their optical properties [41, 59], owing to the synergistic effect of monometallic nanostructures [60].

In this work, gold and silver nanoparticles were synthesized using as a reductor agent a *Rumex hymenosepalus* extract, which is a plant that contains stilbenes and catechins molecules that act as powerful antioxidants in reducing metal ions. Gold nanoparticles were used as nuclei to obtain bimetallic nanoparticles core@shell type of Au@Ag through a sequential synthesis method. The characterization of nanomaterials involves the techniques of HAADF-STEM, TEM with EDS y HRTEM.

With the different types of synthesized nanoparticles, a comparative study was carried out on the growth dynamics of the bacteria *E. coli* and *S. aureus* and the yeast *Candida albicans.*

## Experimental Section

### Materials

The roots of *Rumex hymenosepalus* were acquired in a commercial establishment of the locality. Both precursors HAuCl_4_ and AgNO_3_ for nanoparticle synthesis were purchased from Sigma-Aldrich with a purity of 99%. Brain Heart Infusion (BHI) Broth and Potato Dextrose Broth (PDB) used for microorganism assays were procured from Sigma-Aldrich. Ethanol (99% pure) used in the cleaning nanoparticles process was acquired from Fermont, and ultrapure water (milli-Q) was used in experiments.

### Rumex hymenosepalus Extract

For extract preparation, 150 g of root cut into slices and previously dehydrated were macerated in 1000 mL of a 70/30 V/V ethanol/water mixture. The maceration was carried out at room temperature in a glass container with a hermetic stopper and protected from light. The absorbance of the solution obtained was monitored for 21 days until there were no changes in its value. At this time, the maceration process was considered finished. The extract obtained was successively filtered with Whatman paper filters with pore sizes of 8 µm and 2 µm and finally with an acrodisc filter of 0.22 µm. Ethanol was removed by rotovaporation, and the aqueous extract concentrate was frozen at -80 °C for lyophilization (Labconco, FreeZone 1L). The powders obtained were stored in sterile containers protected from light at room temperature until use.

### Synthesis of AuNPs

Firstly, *Rumex hymenosepalus* aqueous solution was prepared at 10 mg/mL from the lyophilized extract. Later 16 mL of *Rumex* solution was mixed with 32 mL of ultrapure water, keeping agitation at 1000 rpm and were added slowly 16 mL of HAuCl_4_ (0.01 M). The reaction was held for one hour under laboratory lighting conditions and at room temperature. The intensity of surface plasmon resonance (*λ*_SPR_ = 540 nm) was evaluated by UV–Vis spectroscopy over time; when no change was observed, the synthesis was considered complete. The obtained product was centrifuged at 12,000 rpm, the supernatant was replaced by ultrapure water, and a sonication process was applied for 1 h to redisperse nanoparticles. The procedure was repeated three times more. Water was used as a solvent on the first two occasions and ethanol on the last. Finally, ethanolic dispersion was centrifuged, and precipitates dried in a convection oven at 40 °C to prepare an AuNPs aqueous dispersion at 2300 µg/mL.

### Synthesis of Au@AgNPs

For Au@AgNPs synthesis, 2 mL of the AuNPs aqueous dispersion (2300 µg/mL), 0.8 mL of AgNO_3_ (0.1 M), and 0.8 mL of *Rumex hymenosepalus* solution (10 mg/mL) were deposited in a sterile glass culture tube. The mixture was sonicated for 3 h in an ultrasonic cleaner bath (Branson, Model 2510). Later, the content was centrifuged at 12,000 rpm for one hour, solids obtained were redispersed in ultrapure water by sonication to obtain a 1000 µg/mL concentration.

### Synthesis of AgNPs

To produce AgNPs, 16 mL of extract solution (10 mg/mL) was mixed with 64 mL of ultrapure water, and 8 mL of AgNO_3_ 0.1 M. The reaction was carried out for one hour under laboratory lighting conditions at 25 °C, and surface plasmon resonance ($\lambda_{{{\text{SPR}}}}^{{{\text{Ag}}}}$ = 440 nm) was monitored by UV–Vis spectroscopy over time to assess formation. The product was centrifuged at 12,000 rpm, the supernatant was replaced by ultrapure water, and sonication was applied for 1 h. The procedure was repeated three times more. Water was used as a solvent on the first two occasions and ethanol on the last. Ethanolic dispersion was centrifuged and precipitates dried in a convection oven at 40 °C. Finally, AgNPs dust obtained was redispersed in ultrapure water by sonication to generate a colloidal dispersion at 2000 µg/mL concentration.

### Characterizations

UV–Vis absorption spectra were obtained on a dual-beam PerkinElmer Lambda 45 spectrometer. A slite of 0.5 nm was employed, and spectra were recorded at a speed of 480 nm/min in a range between 200 and 900 nm. For the nanoparticles, 50 µL of sample and only 5 µL for the extract were used. The final volume was made up to 3 mL in the quartz cells using ultra-pure water as solvent.

Zeta potential (*ζ*) of AuNPs, AgNPs, and Au@AgNPs were measured using a Zetasizer-Nano ZS (Malvern Instruments, UK). Each sample was measured at room temperature (25 °C) in triplicate and as a function of concentration at 1, 10, 50, and 100 µg/mL for each sample.

DLS for AuNPs, AgNPs, and Au@AgNPs were measured using a Zetasizer-Nano ZS (Malvern Instruments, UK) equipped with a 633 nm He–Ne laser. Each sample was measured at room temperature (25 °C) in triplicate and as a function of concentration at 1, 10, 50, and 100 µg/mL for each sample. Polydispersity Index (PDI) was determined from DLS experiments using Malvern software through the definition ${\text{PDI}} = \left( {\frac{\sigma }{{\overline{D}}}} \right)^{2}$, where *D* is the average Diameter and $\sigma$ is the standard deviation of $D$. PDI values with 0.10 or less are considered highly monodisperse [62].

The optical band gap *E*_g_ was calculated for AgNPs, AuNPs, Ag@AuNPs, using Tauc equation determine a bandgap in materials by the following relationship:

1 $$\alpha = \frac{c}{h\upsilon }\left( {h\upsilon - E_{{\text{g}}} } \right)^{1/n}$$

where $\alpha$ is the absorption coefficient of the material ($\alpha$ = 2.303 *A*/*d* where A is absorbance and d is the width of the cell) and where *E*_g_ is the optical energy band gap and *hʋ* is the photon energy obtained by drawing a line between (*αhʋ*)^*n*^ and photon energy *hʋ*. The index number n is taken as 2 for the allowed direct band to band transitions in samples [63] and can be readily evaluated with a linear fit plot [64]. Depends upon the type of the transition which may have values 1/2, 2, 3/2, and 3 correspond to the allowed direct, allowed indirect, forbidden direct, and forbidden indirect transitions, respectively [65].

Samples AgNPs, AuNPs, Ag@AuNPs, and Rh were analyzed through infrared absorption using an FTIR (PerkinElmer, Inc., Waltham, Spectrum Two). The acquisition parameters were: 4 cm^−1^ resolution, 16 scans, and between 4000 and 900 cm^−1^ wavelength range.

X-ray photoelectron spectroscopy assays were carried out on a PerkinElmer model PHI 5100, which contains a dual source of Mg/Al, 300 W, 15 kV. The Mg Kα emission line with the energy of 1253.6 eV was used for AgNPs, AuNPs, Ag@AuNPs, and Rh. All experiments were performed under vacuum conditions of 2 × 10^–9^ Torr. [66]. Data were analyzed using Multipak software. For XPS characterization, the different nanoparticle dispersions and the *Rumex hymenosepalus* extract solution were deposited on clean coverslips as follows. 30 µL of the sample was added to the coverslip allowing it to dry completely for the next deposit. The process was repeated at least 5 times until a thin film was formed and then characterized by XPS.

High-Angle Annular Dark Field-Scanning Transmission Electron Microscopy (HAADF-STEM) can be considered a powerful operation mode in electron microscopy, which provides a large amount of complementary information elucidates the structure of a nanomaterial. Aberration-corrected HAADF-STEM can determine with atomic resolution the positions of atoms of different chemical nature. This is due to aberration-corrected HAADF-STEM, known for its chemical sensitivity and high spatial resolution [67]. In this operation mode, the incoherent image is dominant with a negligible contribution of diffraction contrast [68]. Thus, atomic number contrast (Z contrast) in HAADF-STEM aberration-corrected allows us to determine the structural details of nanostructures with great precision.

For STEM analysis, samples were analyzed in a JEOL-JEMARM200 electron microscope operating at 200 kV, with a CEOS-corrector for the condenser lens. Z-Contrast STEM images were recorded simultaneously in both BF and HAADF modes. Images were recorded with a 40-micron condenser lens aperture (32–36 mrad convergence angle) and a spot size of 9 pA.

For electron microscopy analysis, a drop (10 µL) of Au@AgNPs suspension was deposited on a 300-mesh thick carbon grid, dried to room temperature, and placed in a vacuum chamber for 24 h.

Nanoparticles were analyzed by TEM in Jeol 2010F apparatus (1.9 Å resolution) at 200 kV. EDS analysis was realized using a QUANTAX 200-TEM X-ray spectrometer (Bruker) with XFlash 4010 detector. For HRTEM analysis, TEM micrographs were recorded at magnifications greater than 100,000X. Interplanar spacings of crystal planes were determined by digital micrograph analysis (3.0 Gatan Version). Sample preparation was similar to that described above for STEM analysis.

Data were collected using a Bruker D8 QUEST diffractometer system equipped with a Multilayer mirrors monochromator and a Cu Kα Microfocus sealed tube (*λ* = 1.54178 Å). Frames were collected at *T* = 300 K via *ω*/*φ*-scans and then processed to obtain diffractograms of Intensity vs. 2Theta. High Score Plus software was used for raw data treatment and the ICSD powder diffraction database associated with software was implemented for the search-match phase identification analyses.

### Antibacterial Activity Assay

Microorganisms tested were bacteria *Escherichia coli* (ATCC 25922), *Staphylococcus aureus* (ATCC 5538P), and yeast *Candida albicans* isolated from infected urine collected from an adult male patient with urinary tract infection (our study follow the principles of the Declaration of Helsinki). Brain Heart Infusion (BHI) and Potato Dextrose Broth (PDB) were used to prepare inoculum of bacteria and yeast, respectively. Cultures were incubated at 37 ºC overnight. The concentration in colony-forming units (CFU/mL) per milliliter of suspension was determined by measuring the optical density by UV–Vis at 540 nm for bacteria, 600 nm for yeast. Suspensions containing 4 × 10^8^, 7.8 × 10^8^, and 2.5 × 10^6^ CFU/mL were used for *E. coli*, *S. aureus*, and *C. albicans*, respectively. Antimicrobial activity was tested in 96-well plates, using liquid culture medium added with nanoparticles and microorganism at 37 ºC. All tests were performed by triplicate. Absorbance was measured in a multimode plate reader Synergy HTX Biotek, using Gen 5 software. All the microbial growth curves were performed using the Origin Lab 8.0 software. As a first step, 70 µL of fresh broth medium (BHI or PDB, according to the studied microorganisms) mixed with nanoparticles at the required concentration were added to the wells. Later, 30 µL of microorganism suspension was added to the wells and homogenized with the medium. The final volume on each well was 100 µL. After dispensing the inoculum, the 96-well plates were read in the spectrophotometer described above. Plates were kept at 37 °C for 24 h, with a circular shaking mode before each reading with a period of 15 min each. The growth rate of microorganisms was determined by optical density measurement (OD) at the mentioned wavelength.

### Curves Growth Analyzed by the Gompertz Model

It is well known in the literature that the Gompertz growth model well describes the growth of microorganism populations. This model allows us to understand two critical parameters in the description of the growth of the microorganism population: the adaptive phase (Lag phase) and the population growth rate. In particular, studying the behavior of the Lag phase in inhibitory treatments of microorganisms is relevant because it provides information on the adaptive responses of the microorganism to the treatment and can even give indications about the development of resistance of the microorganism to the evaluated treatment [69].

The modified Gompertz model has been described by Zwietering et al. [70] and adapted by Li et al. [69] and other authors [71–78] as a model that adjust the growth curves and

2 $$y = A\exp \left\{ { - \exp \left[ {\frac{\mu e}{A}\left( {\lambda - t} \right) + 1} \right]} \right\}$$

where *A* is the cell number expressed as OD_540_ (*S. aureus* and *E. coli*) and OD_600_ (*C. albicans*), *μ* is the growth rate at the exponential phase and *e* is the exponential *e*^*1*^, $\lambda$ is the lag phase. We have adjusted our growth kinetic using a software origin 9.1 to analyze the effects over *S. aureus*, *E. coli,* and *C. albicans* of the three agents AuNPs, AgNPs, and Au@AgNPs.

### Effect of Nanoparticles over *E. coli,*, *S. aureus*, and *C. albicans* Growth

*Escherichia coli* and *Staphylococcus aureus* were inoculated in BHI medium, and *C. albicans* was inoculated in PD broth. All cultures were incubated overnight at 36° C. After incubation, the three cultures were adjusted to absorbance of 1, 0.7, and 1, respectively (*λ* = 540 nm). AuNPs, AgNPs, and Au@AgNPs were adjusted to a concentration of 50 µg/mL. In a 96 microwell plate, adjusted cultures were exposed to nanoparticles in a relationship 7:3, reaching a final volume of 200 µL each microwell. Also, adjusted cultures were exposed to sterilized water as a control condition in the same previously described conditions. Others used controls for this experiment were fresh media and each nanoparticle solutions without microorganisms. *Escherichia coli, Staphylococcus aureus*, and *Candida albicans* were exposed to AuNPs, AgNPs, and Au@AgNPs, respectively. Microwell plate was incubated at 36° C, and a sample of each microorganism in treatment and control conditions was obtained at 1, 7, 13, and 19 h. Collected samples were diluted in serial 1:10 saline solution and spread over Müeller–Hinton plates surface. Inoculated plates were incubated at 36 °C for 24 h. After incubation, CFU/mL were calculated using direct counting.

### Minimal Bactericidal Concentration (MBC) Determination

*Staphylococcus aureus*, *Escherichia coli*, and *Candida albicans* were inoculated each one into Müeller–Hinton broth incubated overnight at 36° C and adjusted to 0.5 McFarland Nephelometer. Once inoculums were adjusted for Minimum inhibitory concentration (MIC) determination, a microdilution test was performed. Briefly, a 96-well plate was used for this purpose. 160 µL of an adjusted culture of each microorganism was poured into 5 wells. Solutions of AuNPs, AgNPs, and Au@AgNPs of 1000 µg/mL were prepared. Previous solutions were used for preparing 750, 500, and 250 µg/mL solutions. Pure sterilized water was used as a 0 µg/mL of nanoparticles, and 40 µL of each previous solution was added to 160 µL of adjusted cultures. In this way, each culture was exposed to a final concentration of 200, 150, 100, 50, and 0 µg/mL of each tested nanoparticle. The microplate was incubated at 36° C for 24 h. After that, a sample of each well was inoculated on a surface of Müeller–Hinton and incubated in the same previously described conditions. After incubation, Müeller–Hinton plates were observed searching for any growth signal. The concentration where no growth signals were observed was recorded as MBC value.

## Results and Discussions

### Characterization

**Fig. 1:**
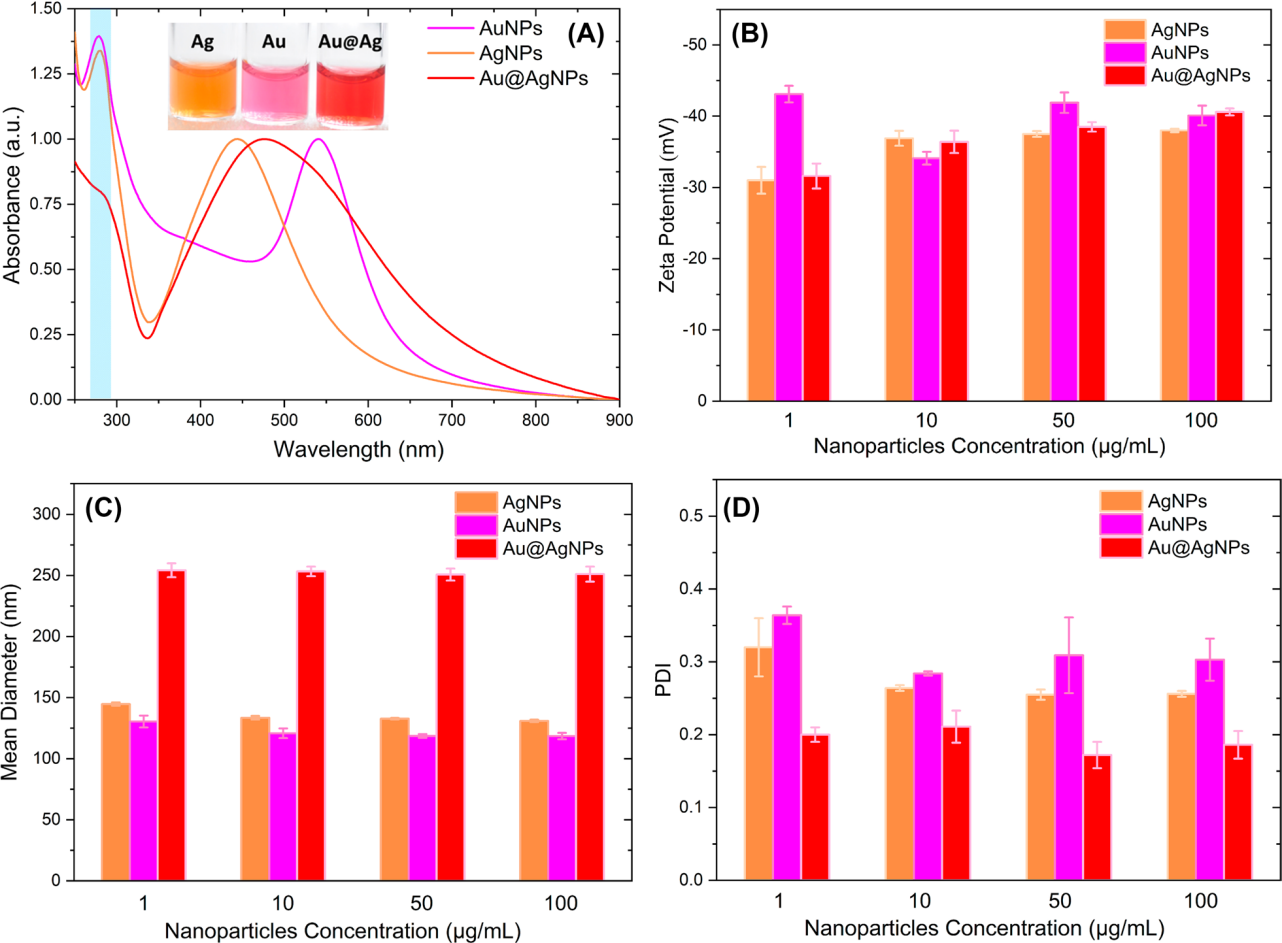
Nanoparticles UV–Vis spectra (**a**), Z-Potential (**b**), DLS (**c**), and PDI (**d**) of Au, Ag and Au@AgNPs

Figure 1a shows the UV–vis absorption spectra of the synthesized nanomaterials. The absorbances have been normalized for the maximum localized surface plasmon resonance (LSPR) corresponding to each nanoparticle system.

Au@AgNps absorption spectrum in Fig. 1a has a single band centered at 474 nm, which is located between the AgNPs LSPR (445 nm) and the AuNPs LSPR (544 nm). The absence of a gold-like absorption peak on Au@AgNPs suggests that obtained nanomaterials by sequential synthesis are core@shell structures. It is not possible to detect any absorption band associated with Au belongs to the nucleus. Some authors assume that for core@shell systems, the absorption spectra are composed of two bands associated with each of the metals for shell thicknesses between 3 and 4 nm. The absorption associated with metalcore disappears for higher thicknesses, obtaining a single absorption band where the maximum location depends on the thickness/core size ratio of the bimetallic particle [79, 80].

Samal et al. [81] synthesized core@shell nanoparticles (Au@Ag) by controlling the nuclei sizes and adding different thicknesses shells. In particular, our UV–vis result for Au@AgNPs coincides with that reported by Samal et al. for 32 nm gold cores and a silver thickness greater than 15 nm, where spectra are characterized by a single absorption band (~ 450 nm), and the suppression of Au surface plasmons is observed.

Additionally, in Fig. 1a, absorption centered at 280 nm (region highlighted in blue) can be observed, corresponding to molecules from the *Rumex hymenosepalus* extract used as a reducing agent in our nanoparticle synthesis. Additional file 1: Figure S1 corresponds to *Rumex hymenosepalus* aqueous solution absorption spectrum. A characteristic band centered at 278 nm is observed, associated with the electronic transitions of the aromatic rings conjugated with the carbonyl groups of polyphenolic compounds [82]. This absorption band in the nanoparticles UV–Vis spectra indicates that final products contain extract molecules that remain in them, Rivero-Cruz et. al. have reported four stilbenoids, two flavan-3-ols, and three anthraquinones isolated from *R. hymenosepalus* [83] and Rodríguez-León et. al. have performed a nuclear magnetic resonance study to determine that *Rumex hymenosepalus* contain important molecules as stilbene glycoside and epicatechin gallate and epigallocatechin gallate [61]. These molecules participate as reducing agents in nanoparticle synthesis. The process involves the deprotonation of some -OH groups of phenolic rings to form = CO groups. Polyphenolic molecules are oxidized, and the released electrons are transferred to Ag^+^ and Ag^3+^ ions to form Au^0^ and Ag^0^.

Figure 1b shows the Zeta potentials values corresponding to AuNPs, AgNPs, and Au@AgNPs at concentrations of 1, 10, 50, and 100 µg/mL. Nanoparticles are dispersed in ultrapure water, and measurement was carried out by triplicate at 25 ℃. In general, throughout the concentration range, nanoparticles exhibit zeta potential values more negative than -30 mV, reaching values of -40 mV at a concentration of 100 µg/mL for AuNPs and Au@AgNPs and -38 mV for AgNPs. These highly negative Zeta potential values indicate that nanoparticles experience repulsive interactions between them that prevent their aggregation and allow the long-term stability of metal colloids [84–86]. By correlating the UV–Vis spectroscopy results with the obtained Zeta potential values, we can establish that the highly negative values may be due to the complexing of polyphenolic molecules of the extract onto the nanoparticle surface [87].

For biological applications, it is valuable to obtain nanoparticles population with monodisperse sizes [88], so Fig. 1c and d shows the DLS values corresponding to mean diameter and polydispersity index (PDI) for AuNPs, AgNPs, and Au@AgNPs at concentrations of 1, 10, 50, and 100 µg/mL. The mean diameter of Au@AgNPs is around 250 nm and kept constant as a function of concentration, in a similar manner for monometallic nanoparticles with a mean diameter around 122 and 135 nm for AuNPs and AgNPs, respectively. In the same case observe that values PDI is around 0.3 for monometallic nanoparticles and 0.2 for Au@AgNPs. These results, where the sizes do not vary with the concentration, indicate that the different nanoparticle systems have good stability presenting a moderate polydispersity of sizes (0.3 ≥ PDI ≥ 0.2).

The optical band gap of Tauc plot was computed (Additional file 1: Figure S2). Conduction band by semiconductors materials have values *E*_g_ < 3 eV, as reference germanium have *E*_g_ < 0.7 eV and silicon is *E*_g_ = 1.1 eV [89]. In our case, the band gap for Au@AgNPs, AuNPs, and AgNPs is 1.93 eV, 2.03 eV, and 2.33 eV, respectively. This means that these materials are considered like semiconductors, and this feature was obtained for the quantum confinement effects that produce an increased energy gap, so nanomaterials can be used as sensors, batteries, and optoelectronic devices [64].

**Fig. 2:**
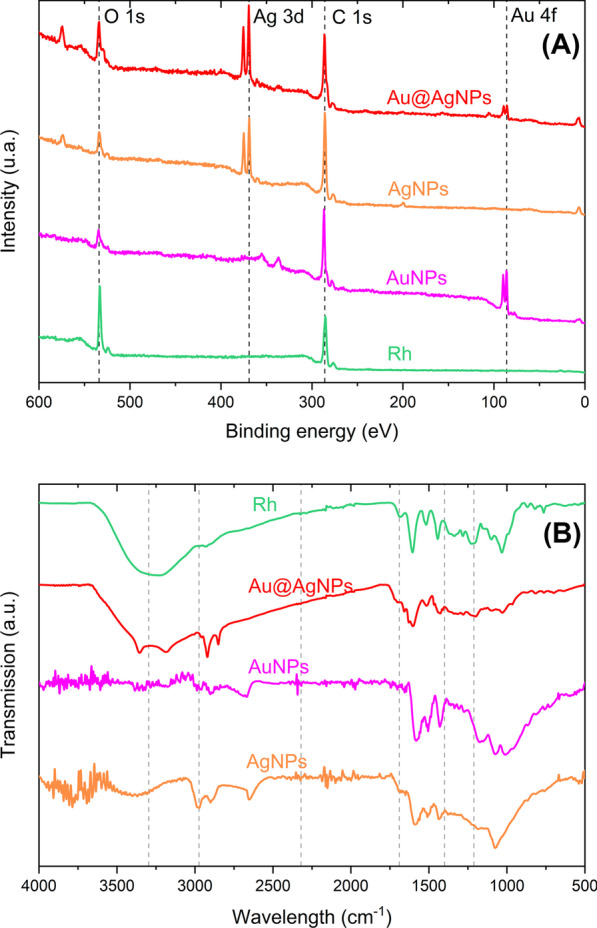
XPS survey spectra (**a**), and FTIR (**b**) for Rh, Ag, Au, and Au@AgNPs

To establish if organic compounds are present in nanoparticles, these nanomaterials were characterized by XPS and FTIR. Figure 2a shows the survey spectra of the plant extract and nanoparticles. As can be seen, all nanoparticle systems present the characteristic signals associated with the elements carbon (C 1 s, 284.6 eV) and oxygen (O 1 s, 532.2 eV) from the *Rumex hymenosepalus* extract. This result confirms that organic molecules from extract are present in nanomaterials obtained. Additionally, high-resolution XPS spectra were obtained to analyze the oxidation states of silver and gold in the nanoparticles (Additional file 1: Figure S3). For AgNPs (Additional file 1: Figure S3A), the signals at binding energies of 373.76 eV and 367.76 eV (∆BE = 6.0 eV) corresponding to the 3d3/2 and 3d5/2 spectral line can be associated with Ag^0^ (metallic silver). For AuNPs (Additional file 1: Figure S3B), the peak associated with 4f5/2 spin-orbital coupling is located on binding energy 87.65 eV. For 4f7/2, spin-orbital coupling peak is located at 83.98 eV. The ratio of intensities (I4f7/2 > I4f5/2), location, and separation between peaks (ΔBE = 3.67 eV) confirm that gold ions (Au^3+^) are reduced completely to metallic gold Au^0^ [90]. For Au@AgNPs, high-resolution XPS spectra corresponding to silver and gold are showed in Additional file 1: Figure S3C and Figure S3-D, respectively. These spectra show the same behavior as their monometallic counterparts. This indicates that both silver and gold are zero-valent in the core@shell presentation.

FTIR in Fig. 2e shows at 3296 cm^−1^ hydroxyl groups, 2974 cm^−1^ (C–H) bind of the aromatic ring, 1689 cm^−1^ associated with carbonyl group stretching (C=O), and 1689–1400 cm^−1^ due to carboxylate bond (C–O) and stretching carbon–carbon bond and 1212 cm^−1^ associated with phenol C–O stretching conjugated with AuNPs, 1049 cm^−1^ C–O stretch mode of the catechin ester group molecules [88–90] and 799 cm^−1^ the bending out of the plane in the phenol, C–H bending reported at 964.4, and 829.39 cm^−1^ feature of resveratrol [91] shifted in Rh extract to 1031 and 867 cm^−1^, respectively, and AuNPs, AgNPs, and Au@AgNPs are shifted too which is indicative of complexation of molecules polyphenolics of Rh extract with nanoparticles.

**Fig. 3:**
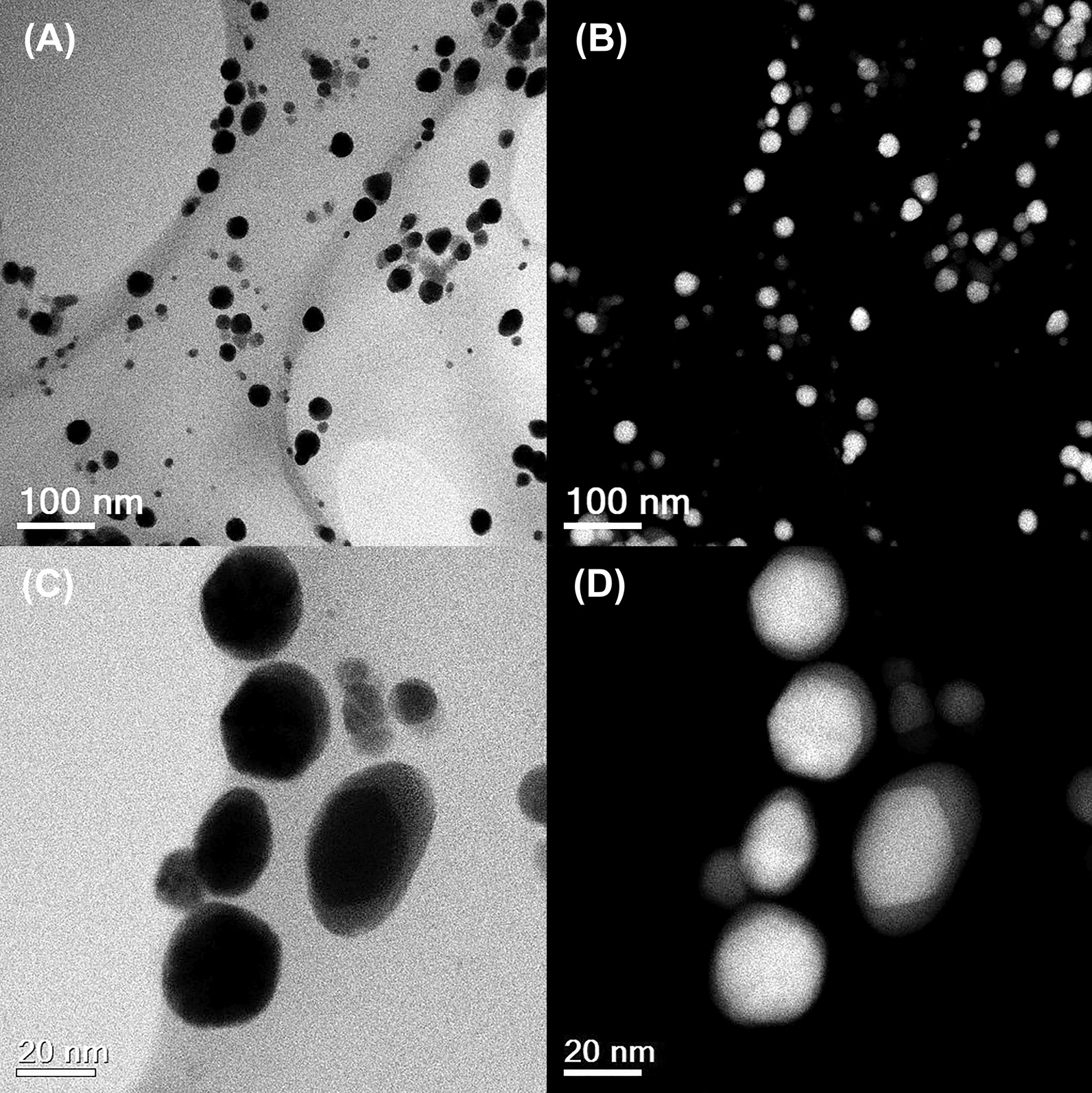
Scanning Transmission Electron Microscopy at low magnification (scale bar 100 nm) of Au@AgNPs in Bright Field (**a**) and HAADF (**b**). A Small group of Au@AgNPs at higher magnification (scale bar 20 nm) in Bright Field (**c**) and HAADF (**d**)

Figure 3a corresponds to a representative bright-field STEM micrograph of the Au@AgNPs system at low magnification (100 nm scale bar). A set of nanoparticles without agglomeration and with mostly quasi-spherical geometry can be seen. The same region is shown in dark field (HAADF) in Fig. 3b, and the core@shell structure can be observed, where can we distinguish Au-core looks more intense than Ag-shell, due to the difference in atomic number. Figure 3c and d corresponds to STEM higher magnification micrograph (scale bare 20 nm) of a nanoparticles group of system core@shell in a bright and dark field, respectively. Can be appreciated with clarity brilliant Au core and Ag shell lightly contrasted. These images show that the thickness of the Ag-shell varies between 3 and 5 nm. Additional file 1: Figure S4 corresponds to an individual images gallery where can be observed uniformity of Ag-shell.

**Fig. 4:**
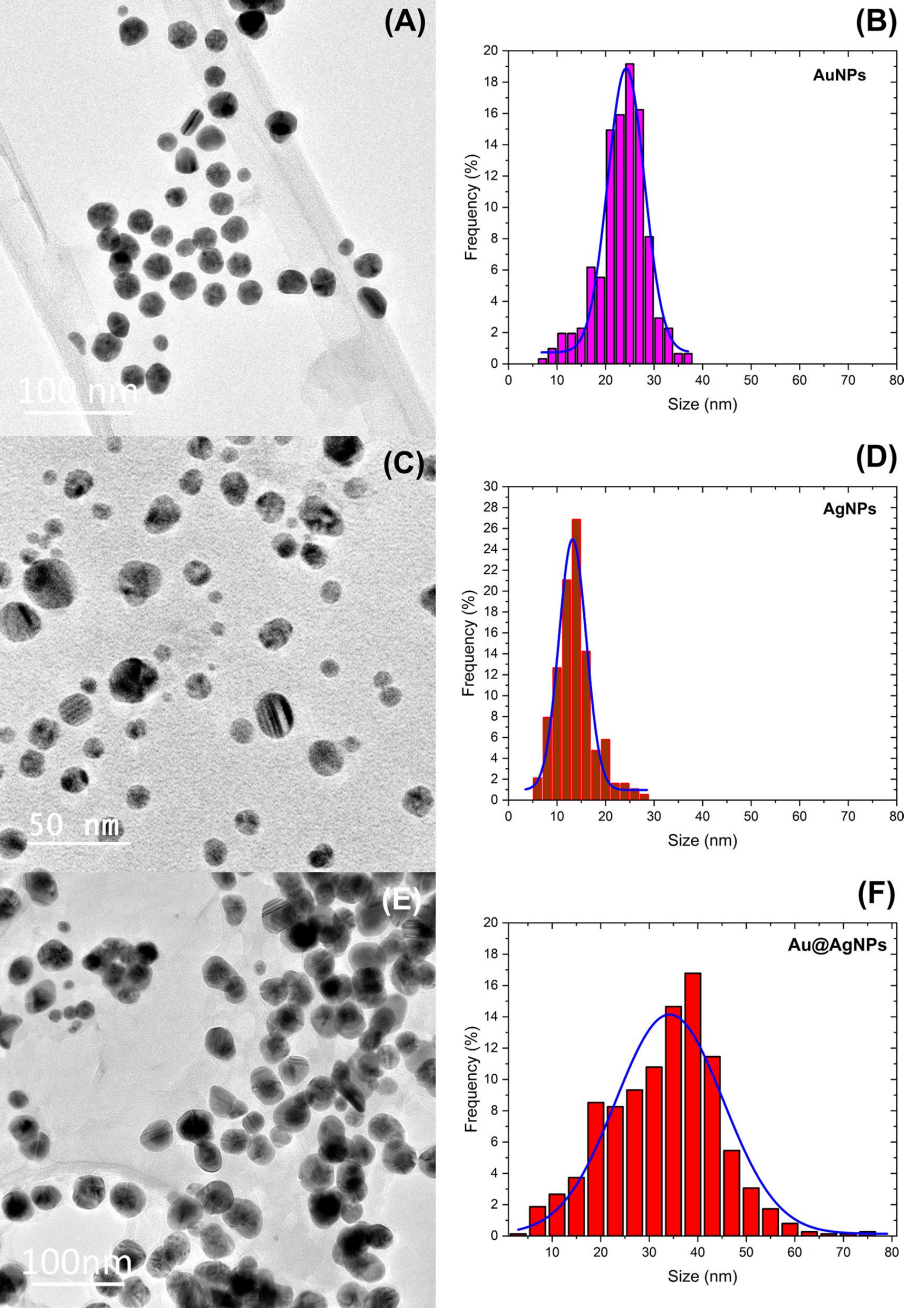
TEM and size distribution of AuNPs in (**a**) and (**b**), AgNPs in (**c**) and (**d**), and Au@AgNPs in (**e**) and (**f**)

Figure 4a, c, and e corresponds to micrograph TEM of representative nanoparticles systems AuNPs, AgNPs, and Au@AgNPs, respectively. In all cases, nanoparticles have sphere-like morphology and are shown well separated from each other. This can be explained by the extract molecules onto nanoparticle surfaces, acting as spacers between them. Figure 4b, d, and f shows histograms corresponding to size distribution obtained by TEM and performed with 500 nanoparticles collected from 15 to 20 micrographs for each nanomaterial. The histogram presents Gaussian distribution with a mean size of 24 ± 4 nm (AuNPs), 13 ± 3 nm (AgNPs), and 36 ± 11 nm (Au@AgNPs). The discrepancy in values between DLS and TEM measurements with size distribution graph is due to conditions micro-environmental around the nanoparticles, while DLS shows a diameter for a system that includes metal, hydrated coating, and solvent by comparison in TEM measurements is a dry system where measurement is over metal only, in particular, in DLS the molecules used for complexation of nanoparticles (reducing agents and stabilizers) are dispersants that induce errors in sizing measurement and shifts it results to higher values [91].

**Fig. 5:**
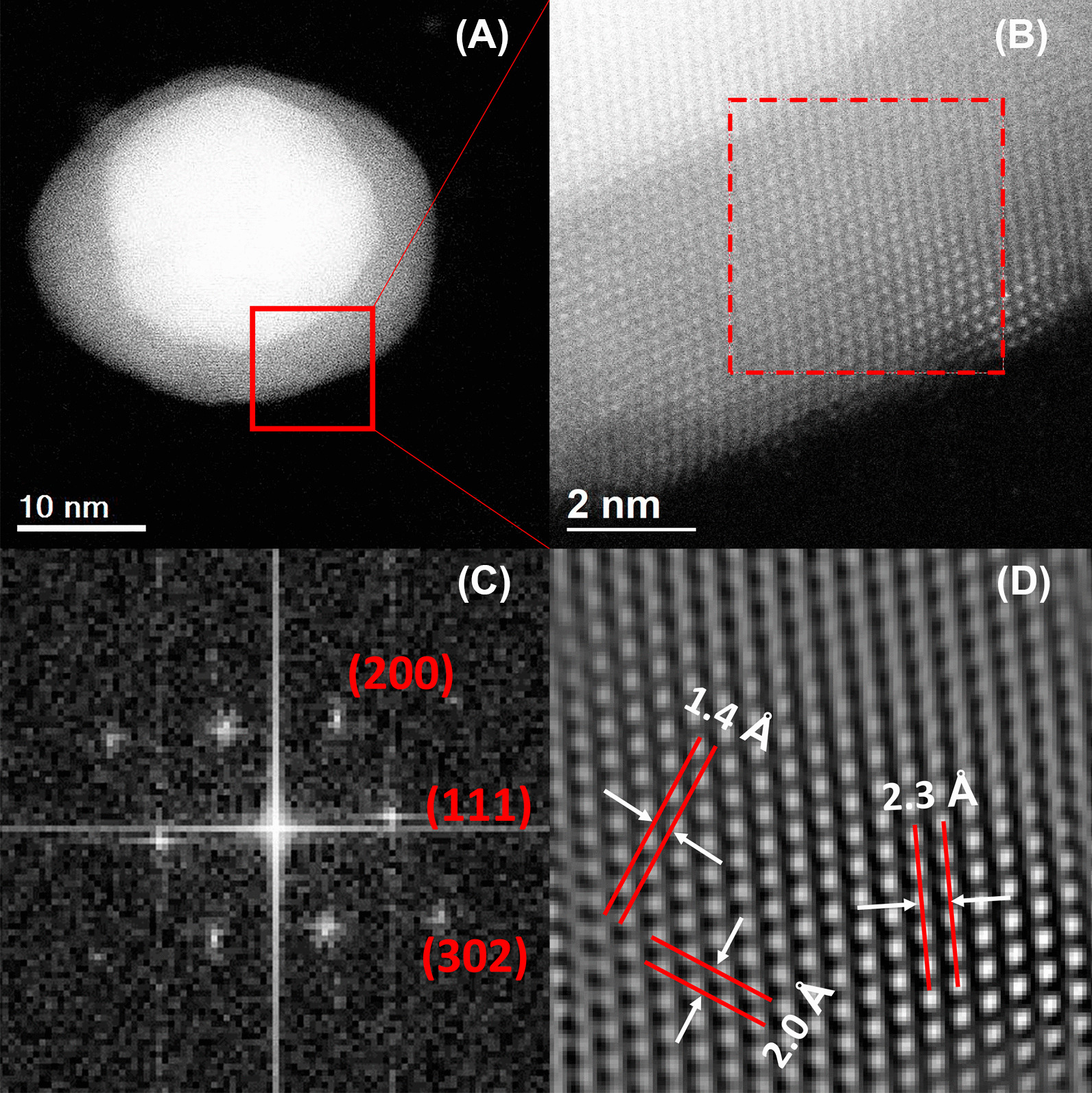
Au@AgNPs HRTEM (**a**). Magnification from (**a**) of interface core–shell (**b**). FFT plot with Miller index (**c**) and integrated image from FFT (Inverse) with interplanar distance (**d**)

Figure 5 corresponds to the Au@AgNPs HAADF-STEM micrographics. A single nanoparticle is shown in Fig. 5a with a gold nucleus and silver cover perfectly delimited, the atomic number (Z) changes through the interface Au@Ag, intensity variations can be quantified by HAADF-STEM [67].

The red square region is amplified to obtain an HRTEM micrography of the shell portion (Fig. 5b), and then to verify the crystalline shell structure, the nanoparticle periphery region was analyzed (discontinued square) with the Digital Micrograph 3.0 software (Gatan). Fast Fourier Transform (FFT) image of the selected area was obtained (Fig. 5c). Using the Inverse Fast Fourier Transform was possible to estimate interplanar distances of 2.3 Å, 2.0 Å, and 1.4 Å in Fig. 5d. These distances can be assigned, respectively, to the crystalline planes (111), (200), and (220) of face-centered cubic (fcc) silver according to Inorganic Crystal Structure Database (ICSD) at the FIZ Karlsruhe–Leibniz Institute for Information Infrastructure, Germany or the electron crystallography software Jems (V 4-5430, JEMS-SAAS, Switzerland) [92]. A similar analysis of crystal structure by HRTEM was carried out for monometallic nanoparticles as illustrated in Additional file 1: Figures S5 (for AuNPs) and S6 (AgNPs). In both cases, crystal structure corresponds to face-centered cubic (fcc).

**Fig. 6:**
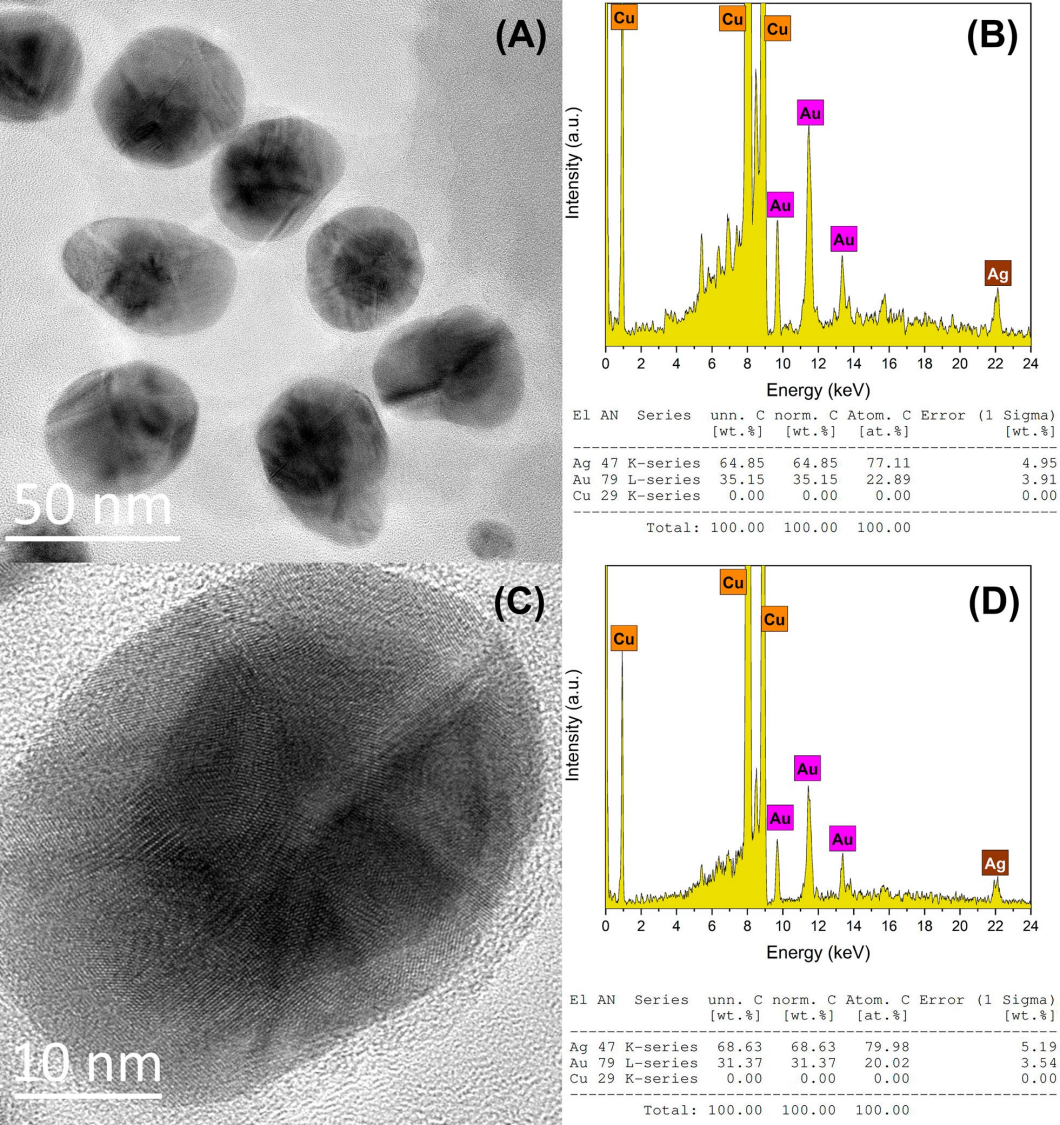
TEM and EDS of an Au@AgNPs group (**a**, **b**) with Au 23 and Ag 77% at. and single Au@AgNPs (**c**, **d**) with Au 20 and Ag 80% at

**Fig. 7:**
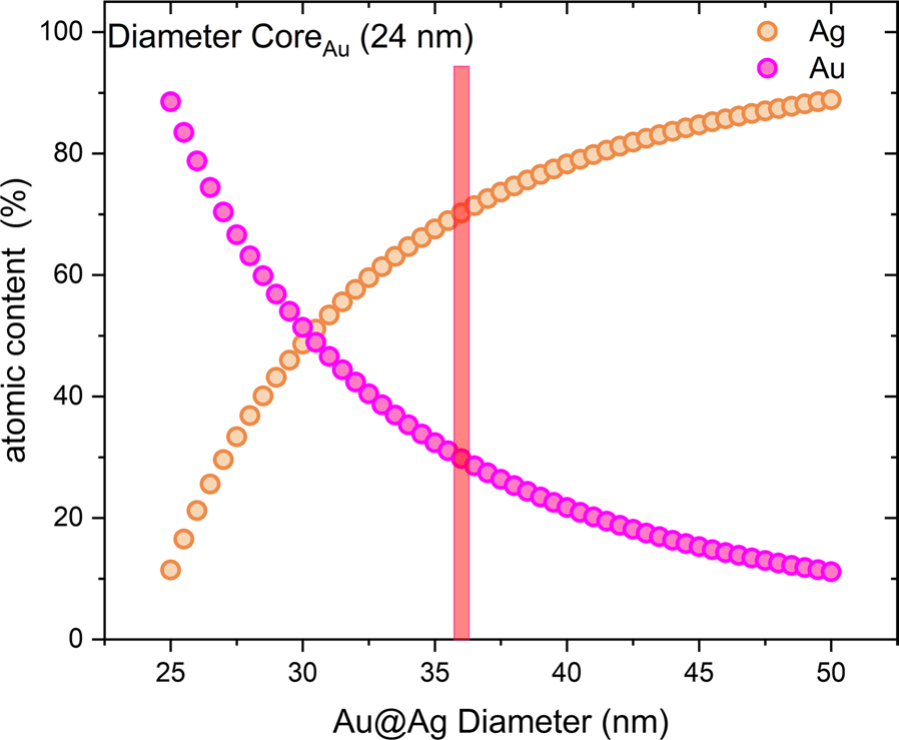
Variations of atomic content percentage versus increase thickness shell, where diameter AuNPs (24 nm) was kept constant

EDS chemical analysis shows the presence of both metals for a group of bimetallic Au@AgNPs observed by TEM (Fig. 6a) in proportions of the atomic weight percent 77% of Ag (shell) and 23% of Au (cores) (Fig. 6b). In comparison, a single bimetallic (Fig. 6c) Au@AgNPs has proportions around 80% of Ag (shell) and 20% of Au (core) (Fig. 6d). To estimate the gold and silver atomic percentage on core@shell nanoparticles (Au@Ag), a quasi-spherical geometry approximation of nanoparticles morphology was considered. The AuNPs average diameter obtained from TEM size distribution ($\overline{D}_{{{\text{Au}}}} = 24\;{\text{nm}}$) was used for core volume estimation (*V*_Au_), and Au@AgNPs average diameter $\left( {\overline{D}_{{{\text{Au}}@{\text{Ag}}}} = 36\,{\text{nm}}} \right)$ for core@shell volume estimation $(V_{{{\text{Au}}@{\text{Ag}}}} )$. So, shell volume is determined as $V_{{{\text{Ag}}}} = V_{{{\text{Au}}@{\text{Ag}}}} - V_{{{\text{Au}}}}$. Total atomic content of Au and Ag was calculated considering an fcc crystalline structure (4 atoms per unit cell) for booth metals where the Au and Ag lattice parameters are 4.0783 Å and 4.0862 Å, respectively [93]. Atomic content estimation by this procedure is 70% for Ag (Shell) and 30% Au (Core), which differs by 10% concerning the measurement obtained by EDS. Figure 7 shows a theoretical estimation of silver and gold content and how varies as the thickness of the shell increases and the size of the core is kept constant ($\overline{D}_{{{\text{Au}}}} = 24\;{\text{nm}}$). It is observed that for Au@AgNPs with a diameter greater than 30.2 nm, the atomic content of silver exceeds the content of gold. In Supplementary Material, a detailed description of calculation is carried out to obtain atomic contents percentage. Similar estimations of atomic percentages were carried out considering other Au-core diameters and varying Au@AgNPs diameter (Additional file 1: Figure S7A-B).

**Fig. 8:**
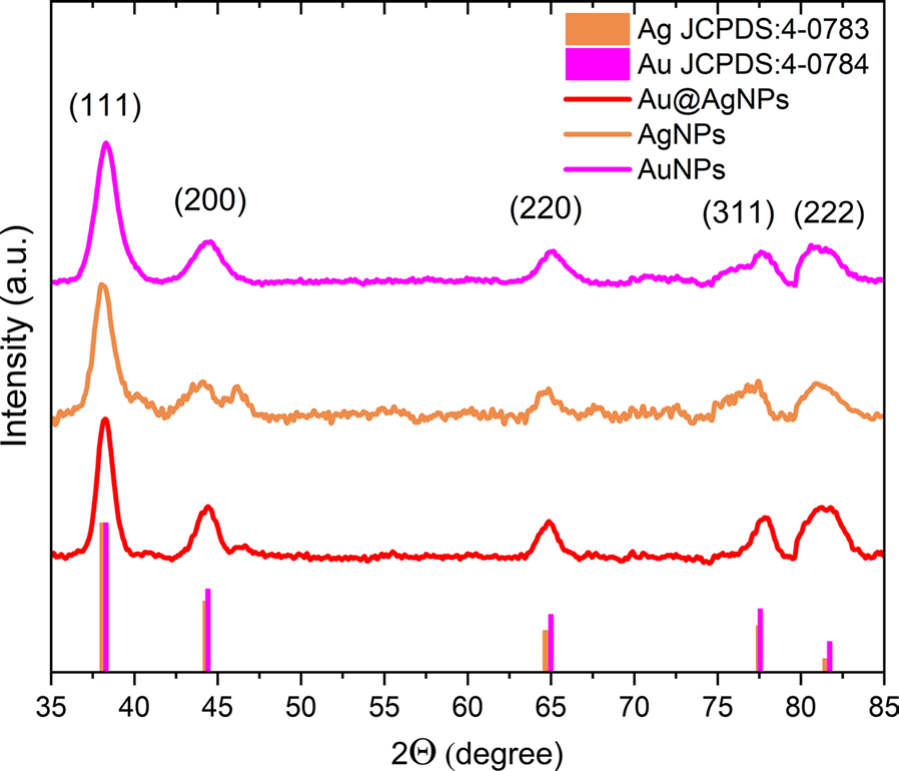
X-ray Diffraction AuNPs (pink), AgNPs (brown), and Au@AgNPs (red)

Figure 8 corresponds to XRD patterns for AuNPs and AgNPs as well as bimetallic Au@AgNPs. All the synthesized products have fcc crystalline structure as previously reported in the characterization by electron microscopy. Peaks for Au@AgNPs are located at 2θ diffraction angles of 38.25°, 44.4°, 64.9°, 77.85°, and 81.25°. As can be appreciated in the figure, the AgNPs and AuNPs diffraction peaks are found in the same positions mentioned with a difference of ± 0.5°. This is because Au and Ag have very similar lattice constants, so their diffraction patterns for fcc crystal structure are almost identical [94–96]. In this way, the diffraction peaks in Fig. 8 are assigned, respectively, to the crystalline planes (111), (200), (220), (311), and (222) of the gold and silver fcc structure by JCPDS: 4-0783 and 4-0784 [97].

### Antimicrobial Activity

**Fig. 9:**
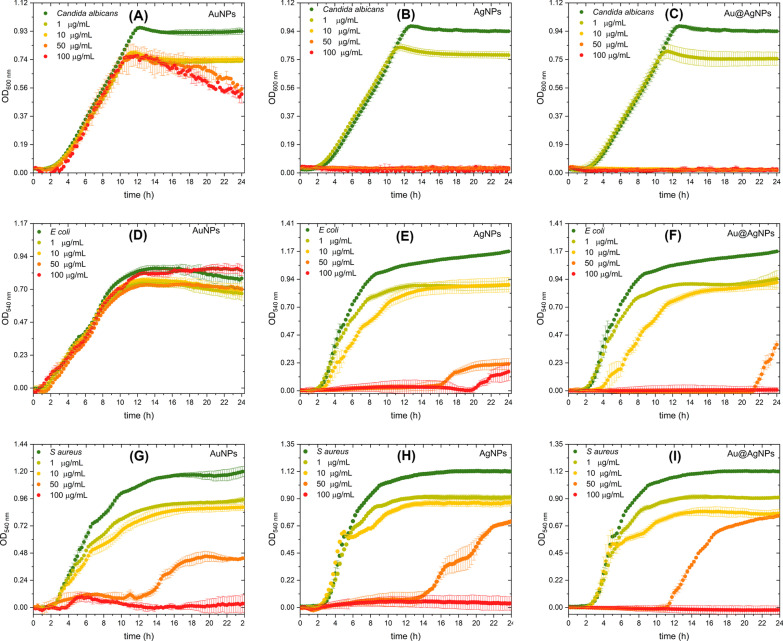
The growth curves using AuNPs, AgNPs, and Au@AgNPs like inhibitory treatments on *Candida albicans* (**a**–**c**), *Escherichia coli* (**d**–**f**), and *Staphylococcus aureus* (**g**–**i**)

Monometallic (AgNPs, AuNPs) and bimetallic (Au@AgNPs) materials were tested at four different concentrations: 1, 10, 50, and 100 μg/mL. Selected microorganisms to evaluate antimicrobial activity were yeast *Candida albicans*, Gram-positive bacteria *S.aureus*, and Gram-negative bacteria *E. coli*. Growth kinetics curves in a time-lapse of 24 h are shown in Fig. 9.

### Candida albicans

AuNPs show no effect on growth kinetics until 10 h (Fig. 9a), varying in a dose-dependent manner the absorbance reached at 24 h. Interestingly, with 50 µg/mL or more, the growth kinetic shows a steep negative slope from 10 h until reaching a 45% reduction at 24 h, which suggests an antifungal effect of these materials. This can be attributed to the ability of gold nanoparticles to interact with relevant proteins present in fungus such as H^+^-ATPase, affecting proton pump activity. This atrophying the ability of yeast to incorporate nutrients causing its death [61]. In Fig. 9b and c was observed that AgNPs and Au@AgNPs inhibit the growth of the yeast *Candida albicans* from 10 µg/mL. The determination of the MIC_50_ concentration for both materials was estimated from the dose–response curve shown in Additional file 1: Figure S8. MIC_50_ is defined as the concentration of nanoparticles that produces a 50% decrease in absorbance concerning the control (yeast without treatment). For AgNPs and Au@AgNPs, MIC_50_ were 2.21 µg/mL and 2.37 µg/mL, respectively.

However, according to the EDS results (Fig. 9b), the silver content in Au@AgNPs is 64.85% mass. Thus, the concentration of silver in Au@AgNPs for MIC_50_ is 1.53 µg/mL, 30% lower than in the case of AgNPs. Padmos et. al. have demonstrated that silver nanoparticles possess important antibacterial features, but are cytotoxic by mammalian cells this effect has reduced using bimetallic nanoparticles especially using gold in the core of bimetallic nanoparticles [35].

### Escherichia coli

In Fig. 9d, AuNPs do not show significant inhibition (< 15%) or affect the growth kinetics of *E. coli*. For AgNPs (Fig. 9e) at low concentrations, the Lag phase remains unchanged, but there is a marked decrease in growth ratio indicated by the slope decrement. At 50 µg/mL Lag phase lasts up to 16 h and viability reaches a maximum of 20% at 24 h. For 100 µg/mL, an apparent detachment of the growth phase of the microorganism is not observed. In Au@AgNPs (Fig. 9f), the first two concentrations do not show changes in their growth phase, but a phase delay of up to 2 h is observed compared to the control. It is interesting to note that the lag phase lasts up to 21 h for the 50 µg/mL concentration, finally there is no explicit growth behavior for the 100 µg/mL concentration.

### Staphylococcus aureus

A comparative analysis of lag phase regrowth occurred after 12 h for Au (Fig. 9g), Ag (Fig. 9h), and Au@AgNPs (Fig. 9i) in the case of *S. aureus* at 50 µg/mL. For the highest concentration at 100 µg/mL, there is no growth of the bacteria. Additionally, we observe changes in the slope of respect control for Au, Ag, and Au@AgNPs at 1 and 10 µg/mL.

AuNPs interaction with these Gram-positive bacteria could be due to the charged surface that causes an electrostatic interaction, destabilizing membrane structure. Similar results, but with higher NPs concentrations, are reported for AuNPs synthesized using Ananas comosus fruit extract as reducing agent [98] and blue-green alga *Spirulina platensis* protein [99]. Yang et al. show MIC > 500 µg/mL for *S. aureus* (CMCC(B)26003), our AuNPs has shown inhibition with 10 times less concentration; in this case, a critical synergy exists with polyphenols molecules on coating and stabilizing the surface of nanoparticle [100]. ROS is generated of less to higher intensity [101] by AuNPs, polyphenols (plant extracts), and AgNPs, so AuNPs in synergy with resveratrol and epigallocatechin gallate (EGCG) promote antibacterial response over *S. aureus* [102] had the most feasible mechanism in this case. Penders et al. reported 250 and 500 µg/mL of AuNPs-like antibacterial agents over *S. aureus* increases in bacterial growth lag time and antibacterial effect [61, 98, 99].

We believe that inhibition is caused by AgNPs [103] accumulation and diffusion on bacteria related to NPs surface charges that promote electrostatic interactions [104] with the bacteria's membrane leading to higher penetration and damage. We think this is a similar mechanism described for interactions between *E. coli* biofilms and AgNps [105].

For Au@AgNPs, the obtained results are comparable to those reported by other workgroups [100, 101]; however, different authors suggest that the inhibition of the growth of the microorganisms is directly related to the thickness of the shell [100, 101]. Core–shell NPs showed low cytotoxicity when tested in NIH-3T3 fibroblasts cells (normal mammalian cells) [35]. A lower proportion of silver in the shell of the Au@AgNPs shows similar results to AgNPs [100], and Au core potentializes antibacterial effect, and minimizes the cytotoxicity.

### Curves Growth Analyzed by the Modified Gompertz Model

**Fig. 10:**
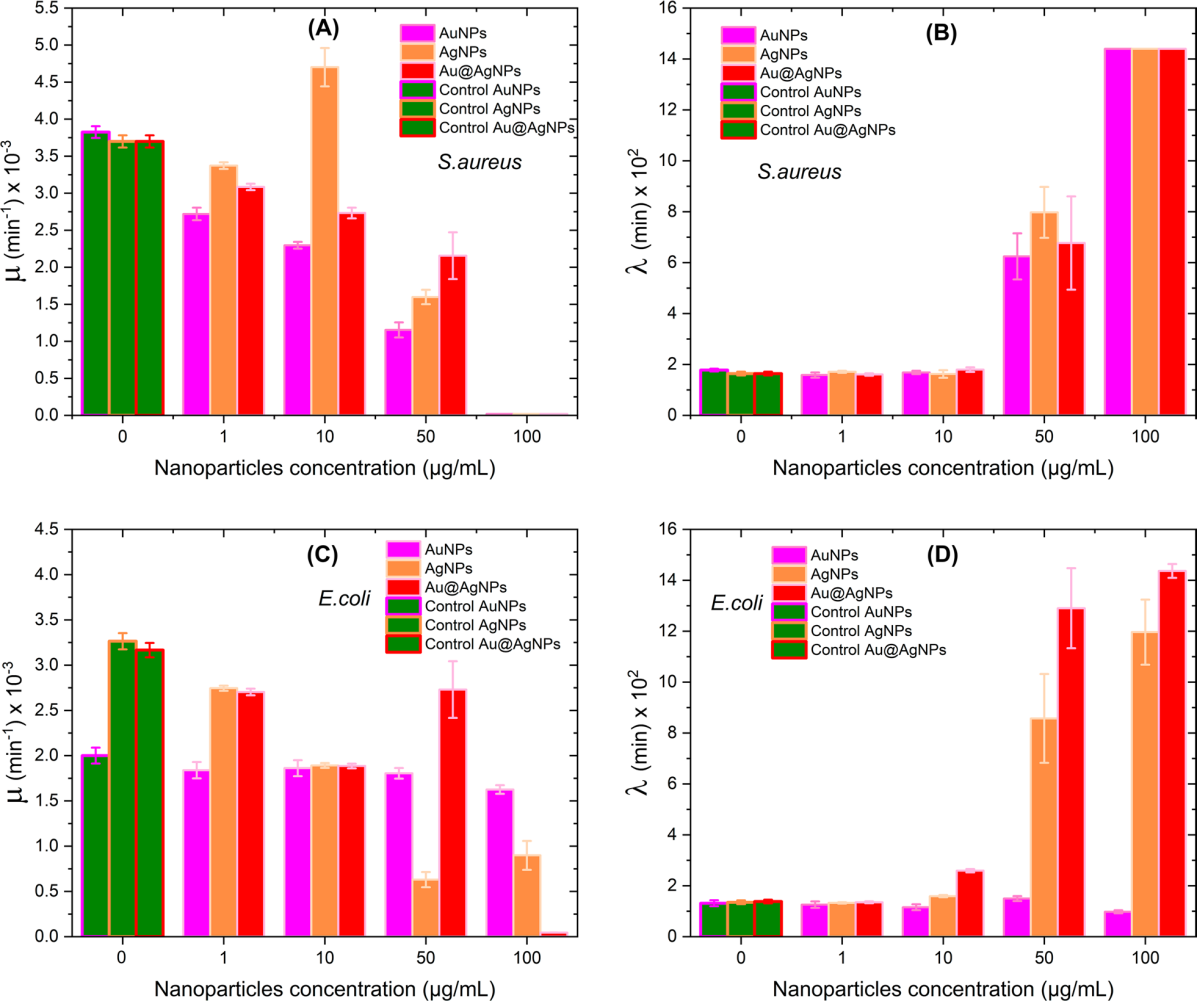
Kinetic parameters were obtained by the Gompertz model. Growth Rate *µ* and Time Lag Phase $\lambda$ for *S. aureus* (**a**, **b**) and *E. coli* (**c**, **d**)

To know how the growth ratios (*µ*) and Lag phase (*λ*) are quantitatively modified, the growth curves of microorganisms exposed to different concentrations of nanomaterials (Fig. 10) were adjusted by the Gompertz model (Eq. 2).

In Fig. 10a, it can be seen that all nanomaterials produce a decrease in the replication rate of *S. aureus* populations when the concentration of nanoparticles increases. This effect results in slightly higher sensitivity for AuNPs. At a concentration of 50 µg/mL, the growth ratio is only 30% concerning control (Additional file 1: Tables S1-S24); at a concentration of 100 µg/mL, all materials inhibit the growth of the *S. aureus* population. The behavior of the adaptive phase for *S. aureus* with the different treatments is shown in Fig. 10b. It is observed that there are no significant differences in the material used, and at 50 µg/mL, the Lag phase has increased by almost 5 times compared to the adaptive phase of *S. aureus* (Additional file 1: Tables S1-S24). In general, we can establish that the different nanomaterials evaluated in *S. aureus* reduce the replication rate and postpone the adaptive phase in a dose-dependent manner until its inhibition at 100 µg/mL.

Figure 10c clearly shows that AuNPs do not affect the growth ratio *µ* of *E. coli* bacteria. Meanwhile, AgNPs produce a decrease over *µ*, reaching a minimum value corresponding to 19% to the control (*µ* for *E.coli* without treatment) for 50 µg/mL (see Additional file 1: Table S52). In contrast, Au@AgNPs completely inhibit the *E. coli* growth at 100 µg/mL. Analysis of the behavior of *E. coli* Lag phase exposed to different materials is shown in Fig. 10d. In this case, unlike Fig. 10b, each material has a characteristic response. Thus, AuNPs do not generate any modification in the adaptive phase of *E. coli*, while AgNPs and Au@AgNPs have a dose-dependent effect on the Lag phase, the latter material standing out. Thus, we can establish that AuNPs have no appreciable effect on *E. coli* bacteria, and Au@AgNPs can inhibit replication and, therefore, indefinitely postpone the Lag phase of *E. coli*. Interestingly, this effect is not achieved for AgNPs even though the net silver content is higher than in Au@AgNPs. This suggests that the core@shell presentation of both metals produces a synergy that favors antimicrobial activity. Feng et. al. have reported an electron compensation phenomena from Au to Ag in core–shell and alloy structures, which derive in enhance the cytotoxicity of nanoparticles but kept it the antibacterial properties, that means, a synergy between Au and Ag are assumed, due to observed differences between the monometallic and bimetallic materials [106], but more research is necessary.

**Fig. 11:**
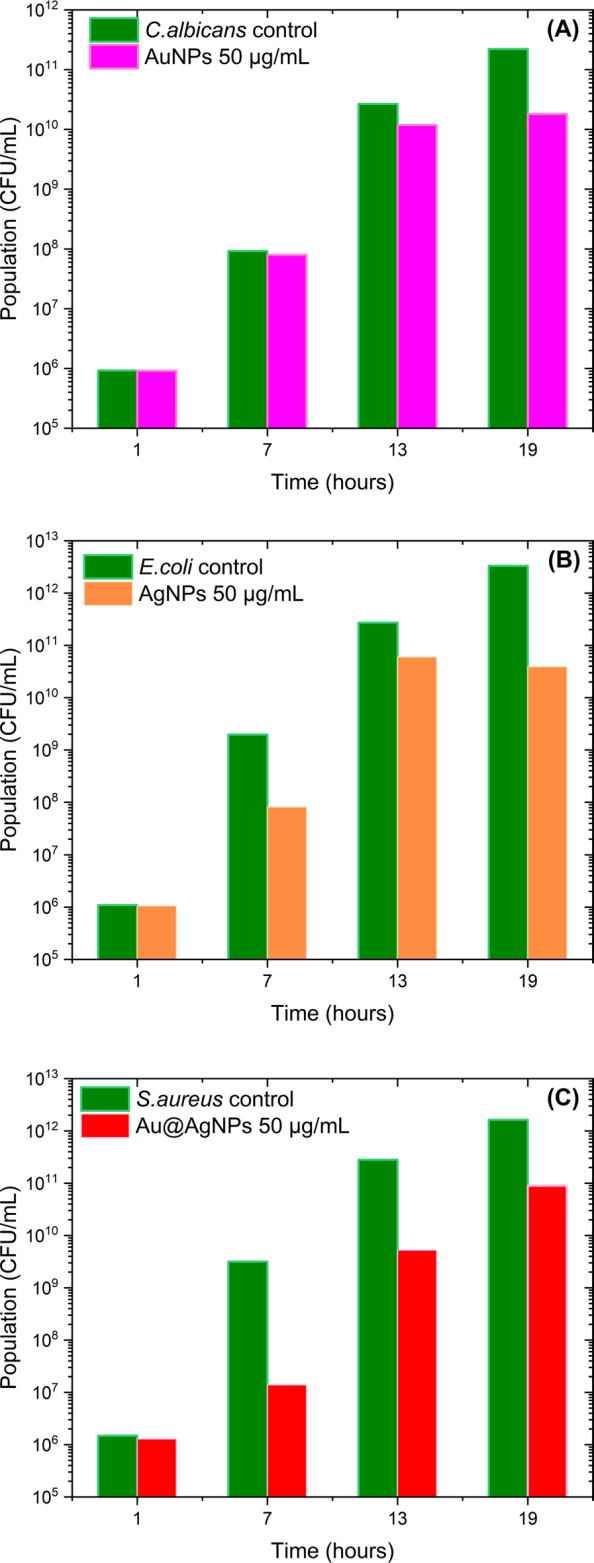
Effect of nanoparticles over microorganisms growth *C. albicans* exposed to AuNPs (**a**), *E. coli* exposed to AgNPs (**b**), and *S. aureus* exposed to Au@AgNPs (**c**). All microorganisms were exposed at 50 µg/mL nanoparticles concentration

Figure 11 shows the results of the direct count study of colonies of microorganisms exposed to nanomaterials. In general, the behavior of the microorganism populations reproduces the results obtained from the growth kinetics study (Fig. 7a, e, i). For example, in Fig. 11a, the population of microorganisms (represented logarithmically) decreases significantly only at 19 h where AuNPs have killed 92% of *C. albicans*. Interestingly, for nanoparticles containing silver (Fig. 11b, c) a more pronounced population decline is observed. Au@AgNPs system at 50 µg/mL, almost entirely inhibits *S. aureus* at 7 h (99.5% of bacteria killed) although microorganism reactivates its growth for later times. MBC determination was not possible to obtain for tested concentrations (Additional file 1: Figure S9-11), according to experimental observation higher concentrations are required to show this effect. However, for *C. albicans*, AgNPs showed an MBC value of 50 µg/mL (Additional file 1: Table S55).

## Conclusions

For the first time, the production of gold nanoparticles and core@shell (Au@Ag) is reported using a *Rumex hymenosepalus* root extract as a reducing agent. To obtain Au@AgNPs is proposed a two-step sequential method that produces particles with moderate polydispersity and homogeneous silver shell. Determination of the growth curves and their parameters obtained through the Gompertz model indicate different effects of the nanomaterials on evaluated microorganisms. Inhibitory effects of AuNPs over *S. aureus* are reached at a concentration of 5 times less to report for other AuNPs synthesized by different processes. This reveals the importance of the synthesis process followed and the environment on the surface of the nanoparticles. On the other hand, AgNPs and Au@AgNPs produce a great growth of the lag phase (> 12 h). However, bacteria can adapt and initiate their growth at these sub-inhibitory concentrations with the consequent risk of generating resistance to these nanomaterials. This highlights the importance of conducting growth kinetic studies that cover an appropriate period to discard a delayed growth. Interestingly, Gompertz's analysis indicates that Au@AgNPs present a higher effect on the growth kinetic of microorganisms than shown by monometallic nanoparticles, which can be attributed to a synergistic effect of both metals on the core@shell structure. Bactericidal effects are only achieved in *C. albicans* exposed to AgNPs. More experiments must be carried out on higher concentration ranges of these nanomaterials (> 200 µg/mL) to determine their MBC on the studied microorganisms.

## Supplementary Information

**Additional file 1**. Figure S1 Corresponds to Rumex hymenosepalus aqueous solution absorption spectrum. Figure S2 A band gap Tauc plot of AgNps, AuNps, and Au@AgNPs. Figure S3 XPS high-resolution for AgNPs, AuNPs, and Au@AgNPs. Figure S4 STEM images gallery of individual Au@AgNPs. The first column corresponds to Bright Field images and the middle column to HAADF. The last column is shown manipulated images using ImageJ to enhance contrast. We can observe the uniformity of the Ag-shell. Figure S5 AuNPs HRTEM (A), region 1 FFT plot (B), and integrated image from FFT (C). Region 2 FFT plot is shown in (D) and integrated image from FFT with interplanar distances in (E). Figure S6 AgNPs HRTEM (A), FFT plot (B) and, integrated image from FFT with interplanar distances (C). Figure S7 Variations of atomic content percentage versus increase thickness shell. Figure S8 C. albicans dose-response curve comparative between Ag and Au@AgNPs. Table S1-S54. Adjust parameters of the curve by modified Gompertz Model (Origin 2018 software) and Physical Parameters of Bacterial Growth obtained with Adjust Parameters for S. aureus, E. coli, and C. albicans at concentrations 0, 1, 10, 50, and 100 µg/mL, respectively. Figure S9 Minimal Bactericidal Concentration determined for E. coli treated with nanoparticles and inoculated in Muller Hinton plate. AgNPs (A), AuNPs (B), and Au@AgNPs (C). Figure S10 Minimal Bactericidal Concentration determined for S. aureus treated with nanoparticles and inoculated in Muller Hinton plate. AgNPs (A), AuNPs (B), and Au@AgNPs (C). Figure S11. Minimal Bactericidal Concentration determined for C. albicans treated with nanoparticles and inoculated in Muller Hinton plate. AgNPs (A), AuNPs (B), and Au@AgNPs (C) Table S55. Minimal Bactericidal Concentration (MBC).

## Data Availability

All data generated or analyzed during this study are included in this article and its supplementary information file.

## Abbreviations

AuNPs: Gold nanoparticles
AgNPs: Silver nanoparticles
Au@AgNPs: Gold–silver bimetallic nanoparticles with core@shell structure
DLS: Dynamic light scattering
EDS: Energy-dispersive X-ray spectroscopy
EGCG: Epigallocatechin gallate
FTIR: Fourier transform infrared spectrometer
HAADF: High angle annular dark field images
MBC: Minimal bactericidal concentration
MIC: Minimum inhibitory concentration
Rh: *Rumex hymenosepalus* Root extract
TEM: Transmission electron microscopy
STEM: Scanning transmission electron microscope
UV-Vis: Spectroscopy ultraviolet–visible spectroscopy
XPS: X-ray photoelectron spectroscopy
XRD: X-ray diffraction
