# Supplementary material for: Au@Ag Core@Shell Nanoparticles Synthesized with Rumex hymenosepalus as Antimicrobial Agent
Source: Nanoscale Res Lett. 2021 Jul 22;16:118. doi: 10.1186/s11671-021-03572-5 (PMC8298724; doi:10.1186/s11671-021-03572-5)
Supplement: Supplementary file 1 — Additional file 1. Figure S1 Corresponds to Rumex hymenosepalus aqueous solution absorption spectrum. Figure S2 A band gap Tauc plot of AgNps, AuNps, and Au@AgNPs. Figure S3 XPS high-resolution for AgNPs, AuNPs, and Au@AgNPs. Figure S4 STEM images gallery of individual Au@AgNPs. The first column corresponds to Bright Field images and the middle column to HAADF. The last column is shown manipulated images using ImageJ to enhance contrast. We can observe the uniformity of the Ag-shell. Figure S5 AuNPs HRTEM (A), region 1 FFT plot (B), and integrated image from FFT (C). Region 2 FFT plot is shown in (D) and integrated image from FFT with interplanar distances in (E). Figure S6 AgNPs HRTEM (A), FFT plot (B) and, integrated image from FFT with interplanar distances (C). Figure S7 Variations of atomic content percentage versus increase thickness shell. Figure S8 C. albicans dose-response curve comparative between Ag and Au@AgNPs. Table S1-S54. Adjust parameters of the curve by modified Gompertz Model (Origin 2018 software) and Physical Parameters of Bacterial Growth obtained with Adjust Parameters for S. aureus, E. coli, and C. albicans at concentrations 0, 1, 10, 50, and 100 µg/mL, respectively. Figure S9 Minimal Bactericidal Concentration determined for E. coli treated with nanoparticles and inoculated in Muller Hinton plate. AgNPs (A), AuNPs (B), and Au@AgNPs (C). Figure S10 Minimal Bactericidal Concentration determined for S. aureus treated with nanoparticles and inoculated in Muller Hinton plate. AgNPs (A), AuNPs (B), and Au@AgNPs (C). Figure S11. Minimal Bactericidal Concentration determined for C. albicans treated with nanoparticles and inoculated in Muller Hinton plate. AgNPs (A), AuNPs (B), and Au@AgNPs (C) Table S55. Minimal Bactericidal Concentration (MBC). [file 11671_2021_3572_MOESM1_ESM.docx]

**Supplementary Material**

Au@Ag Core@shell Nanoparticles Synthesized with *Rumex hymenosepalus* as Antimicrobial Agent

Jesús Mauro Adolfo Villalobos-Noriega^a^, Ericka Rodríguez-León^a*^, César Rodríguez-Beas^a^, Eduardo Larios-Rodríguez^b^, Maribel Plascencia-Jatomea^c^, Aarón Martínez-Higuera^a^, Heriberto Acuña-Campa^d^, Alfonso García-Galaz^e^, Roberto Mora-Monroy^f^, Francisco Javier Alvarez-Cirerol^g^, Blanca Esthela Rodríguez-Vázquez^h^, Roberto Carlos Carillo-Torres^a^, Ramón A. Iñiguez-Palomares^a^*

^a^ Nanotechnology Graduate Program, Department of Physics, University of Sonora, Rosales and Transversal, 83000 Hermosillo, Sonora, Mexico.

^b^Department of Chemical Engineering and Metallurgy, University of Sonora, Rosales and Transversal 83000, Hermosillo, Sonora, México.

^c^Department of Research and Postgraduate in Food, University of Sonora, Rosales and Transversal 83000, Hermosillo, Sonora, México.

^d^Department of Physics, University of Sonora, Rosales and Transversal, 83000 Hermosillo, Sonora, Mexico.

^e^Food Science Coordination, Research Center in Food & Development (CIAD), Road Gustavo Enrique Astiazarán Rosas, No. 46, Col. La Victoria, 83304, Hermosillo, Sonora, Mexico.

^f^Department of Physic Researching, University of Sonora, Rosales and Transversal, 83000, Hermosillo, Sonora, Mexico

^g^ Health Sciences Graduate Program, Department of Biological Chemies, University of Sonora, Hermosillo, Sonora, Mexico

^h^Department of Polymers and Materials Research, University of Sonora, Rosales and Transversal 83000, Hermosillo, Sonora, México.

*Corresponding author: [ramonalfonso.iniguez@gmail.com](mailto:ramonalfonso.iniguez@gmail.com), [ericka.rodriguez@unison.mx](mailto:ericka.rodriguez@unison.mx)

**
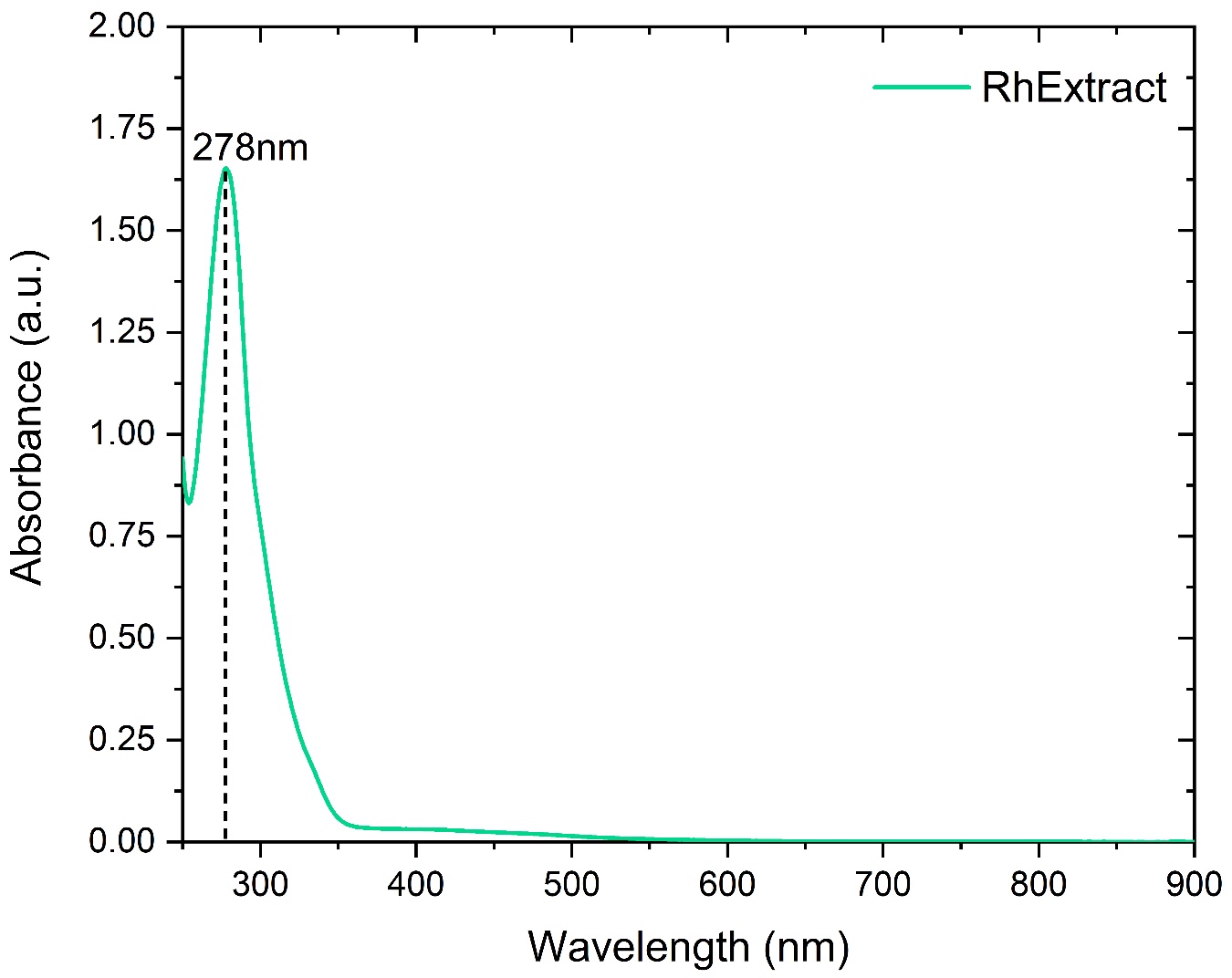
**

**Figure S1** Corresponds to *Rumex hymenosepalus* aqueous solution absorption spectrum.


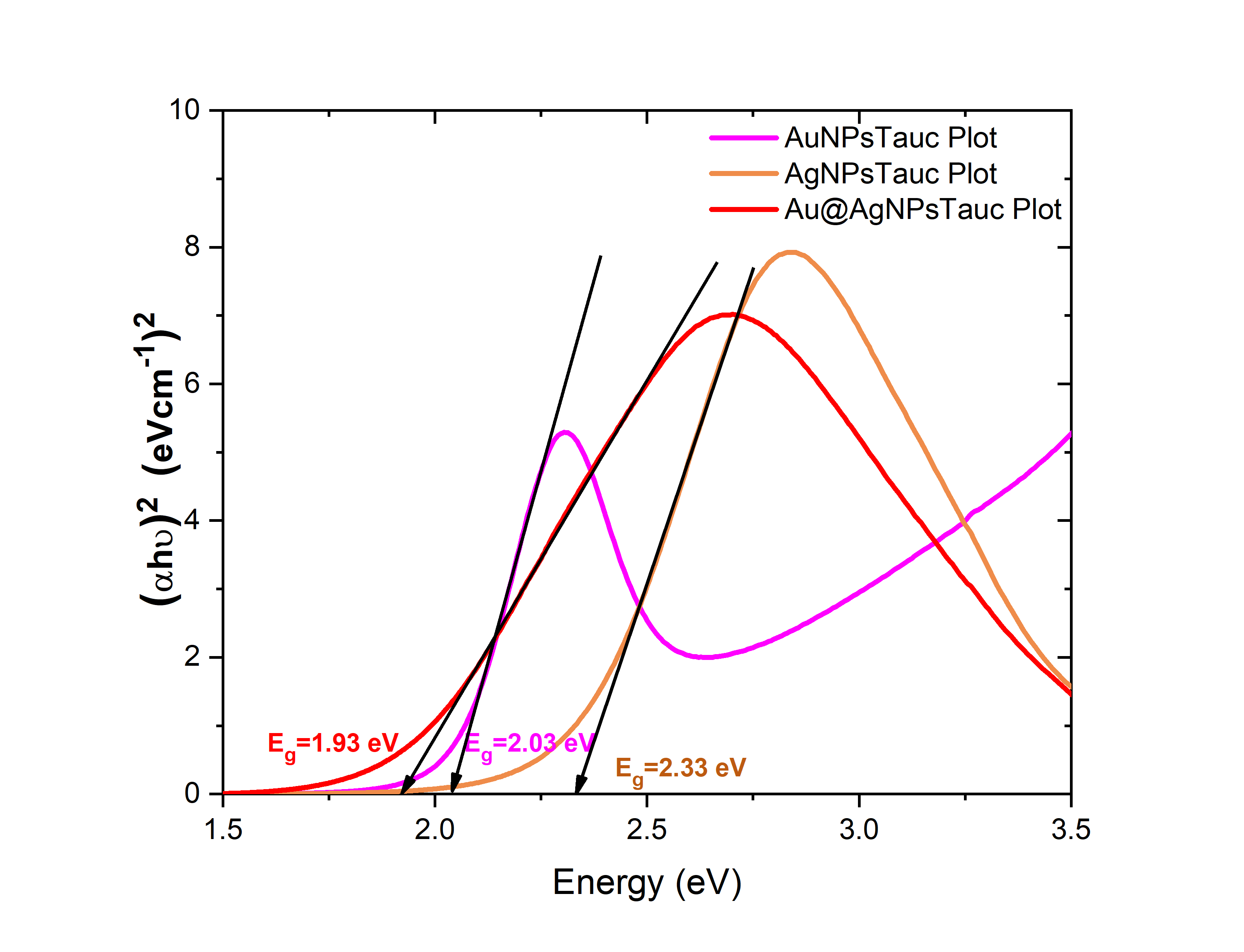


**Figure S2** A band gap Tauc plot of AgNps, AuNps, and Au@AgNPs.

**
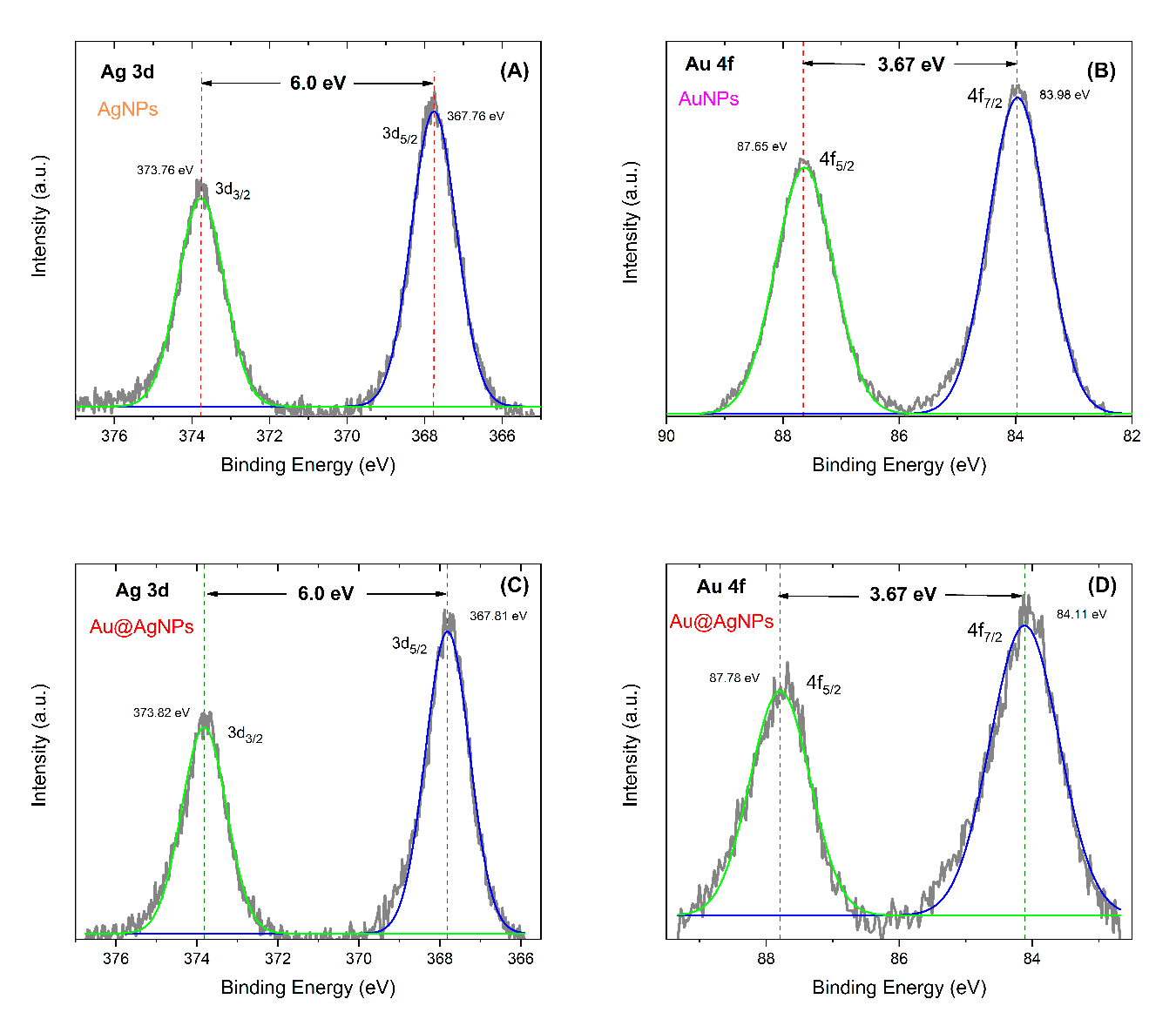
**

**Figure S3** XPS high-resolution for AgNPs, AuNPs, and Au@AgNPs.

**
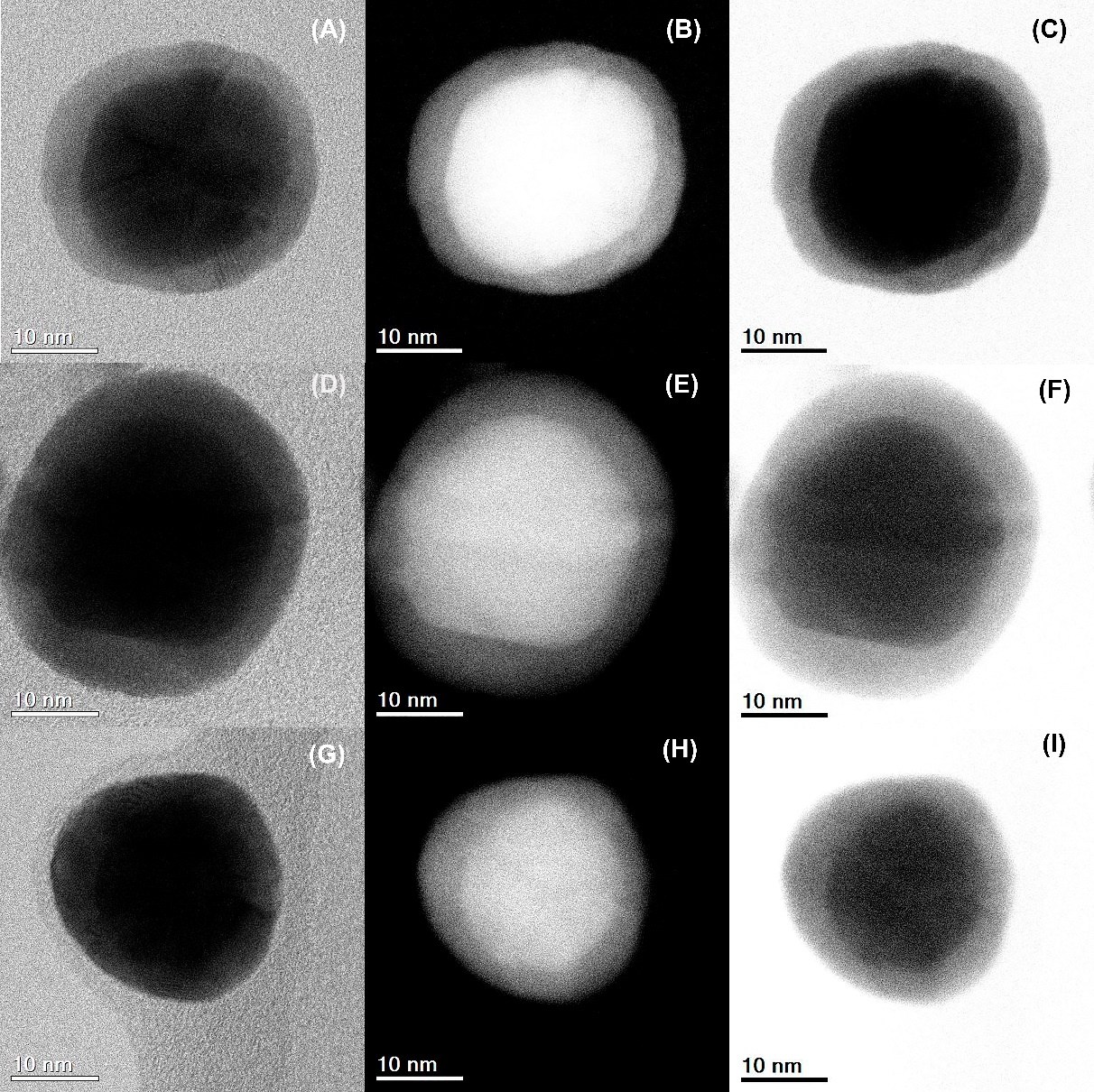
**

**Figure S4** STEM images gallery of individual Au@AgNPs. The first column corresponds to Bright Field images and the middle column to HAADF. The last column is shown manipulated images using ImageJ to enhance contrast. We can observe the uniformity of Ag-shell.


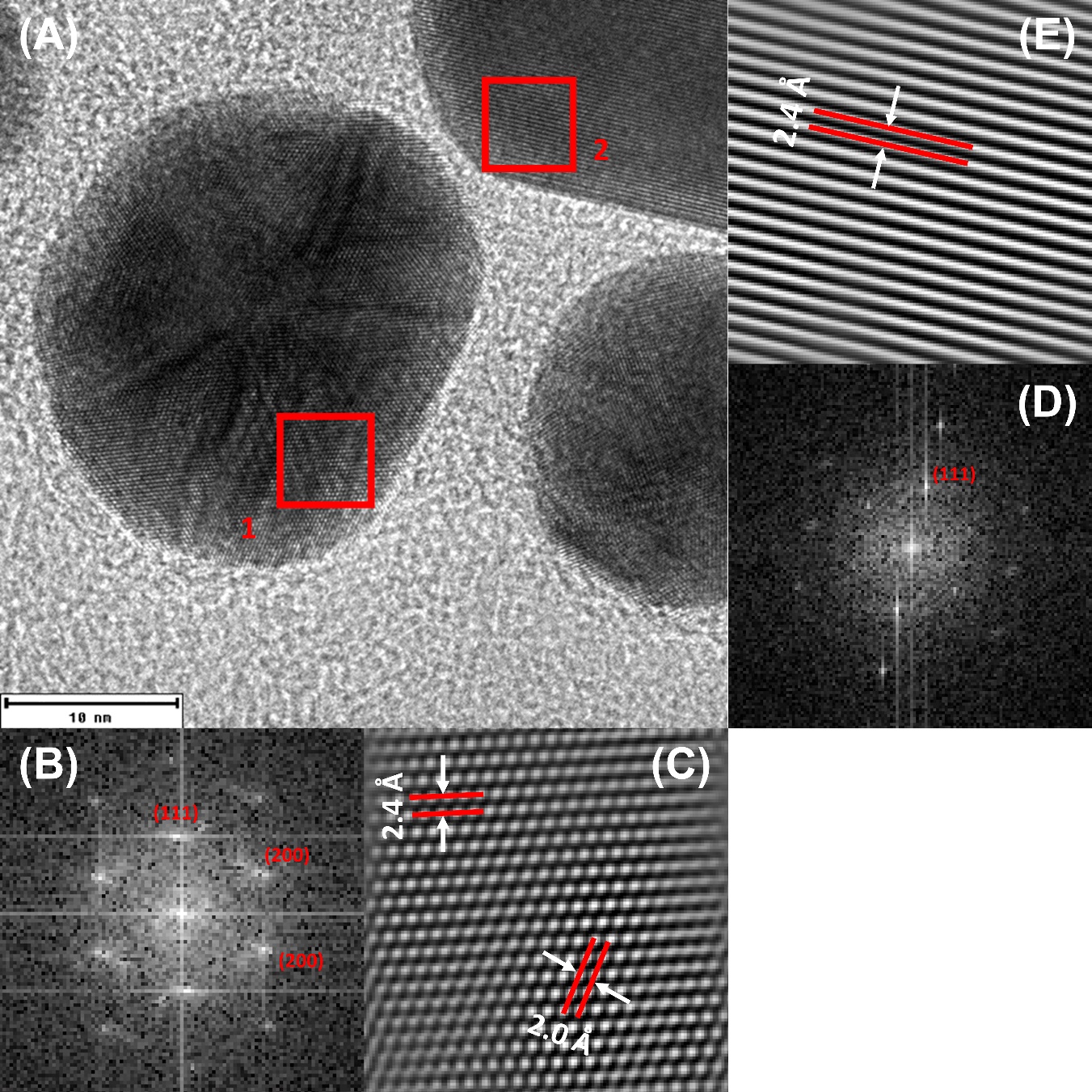


**Figure S5** AuNPs HRTEM (A), region 1 FFT plot (B), and integrated image from FFT (C). Region 2 FFT plot is shown in (D) and integrated image from FFT with interplanar distances in (E).


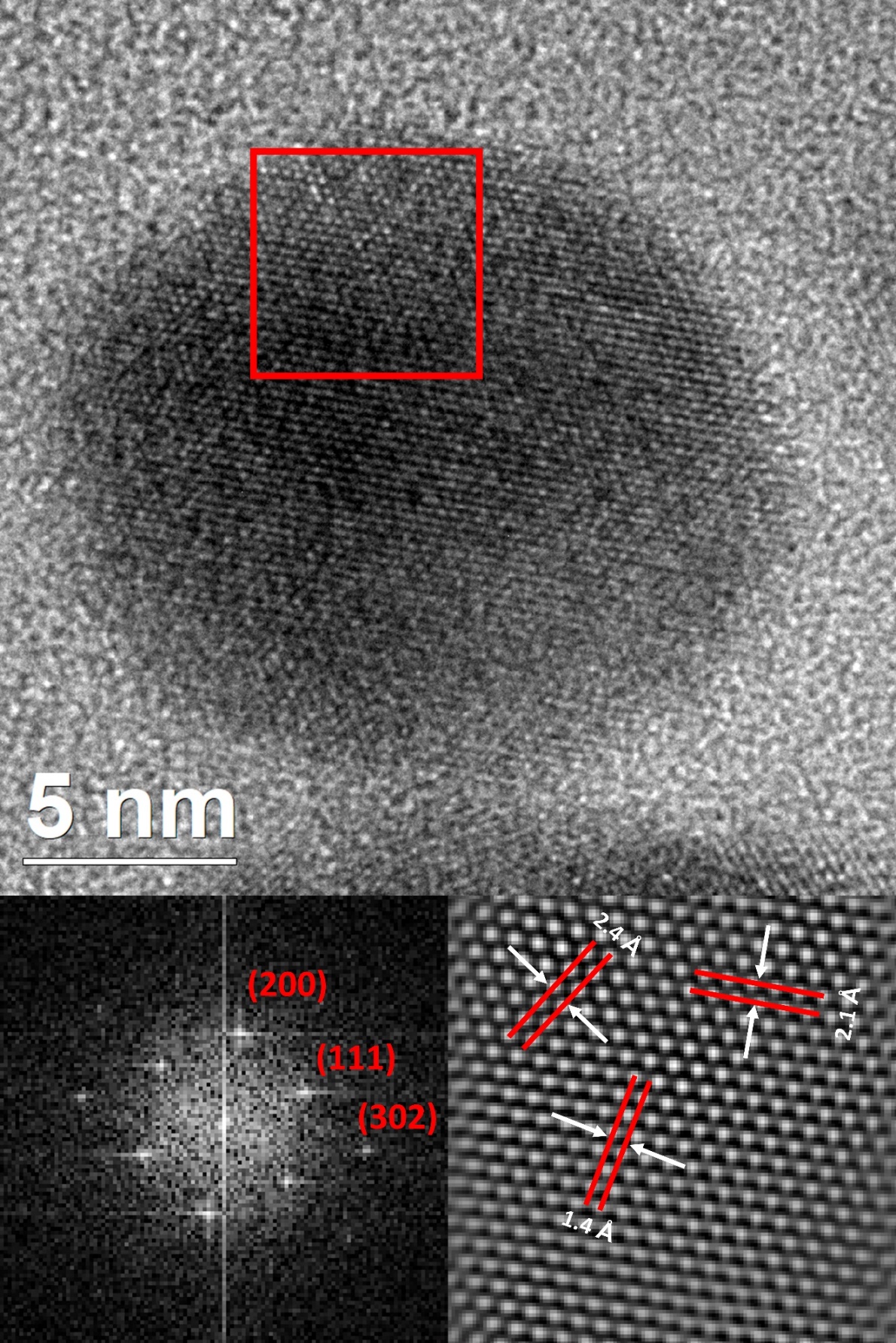


**Figure S6** AgNPs HRTEM (A), FFT plot (B) and, integrated image from FFT with interplanar distances (C).

The atomic content percentage was computed using the values of diameter obtained of TEM, and considering quasi-spherical structures we determined the total volume of Au@AgNPs and the volume of AuNPs for values 20, 24 and 28 nm corresponds with low, mean and high values of diameter. The difference between total volume and AuNPs volume is a mount of silver in Au@AgNPs. Then computed the Total volume of nanoparticle

$$V_{T}=\left( \frac{D_{Au@Ag}}{2} \right)^{3}\frac{4\pi}{3}$$

$$V_{core}^{Au}=\left( \frac{D_{Au}}{2} \right)^{3}\frac{4\pi}{3}$$

$$V_{shell}^{Ag}=V_{T}-V_{core}^{Au}$$

With Volume of cell Au= (0.40783 nm)^3^=0.06783 ${nm}^{3}$ calculus the number of the atoms in AuNPs (core) where there are four atoms by cell (fcc)

Atoms number core Au = $\frac{V_{core}^{Au}}{0.06783 {nm}^{3}}*4$

With Volume of cell Ag= (0.40862 nm)^3^=0.06822 ${nm}^{3}$calculus the number of the atoms of silver shell

Atoms number shell Ag = $\frac{V_{shell}^{Ag}}{0.06822 {nm}^{3}}*4$

Using these data can obtain the atomic content percentage.

**
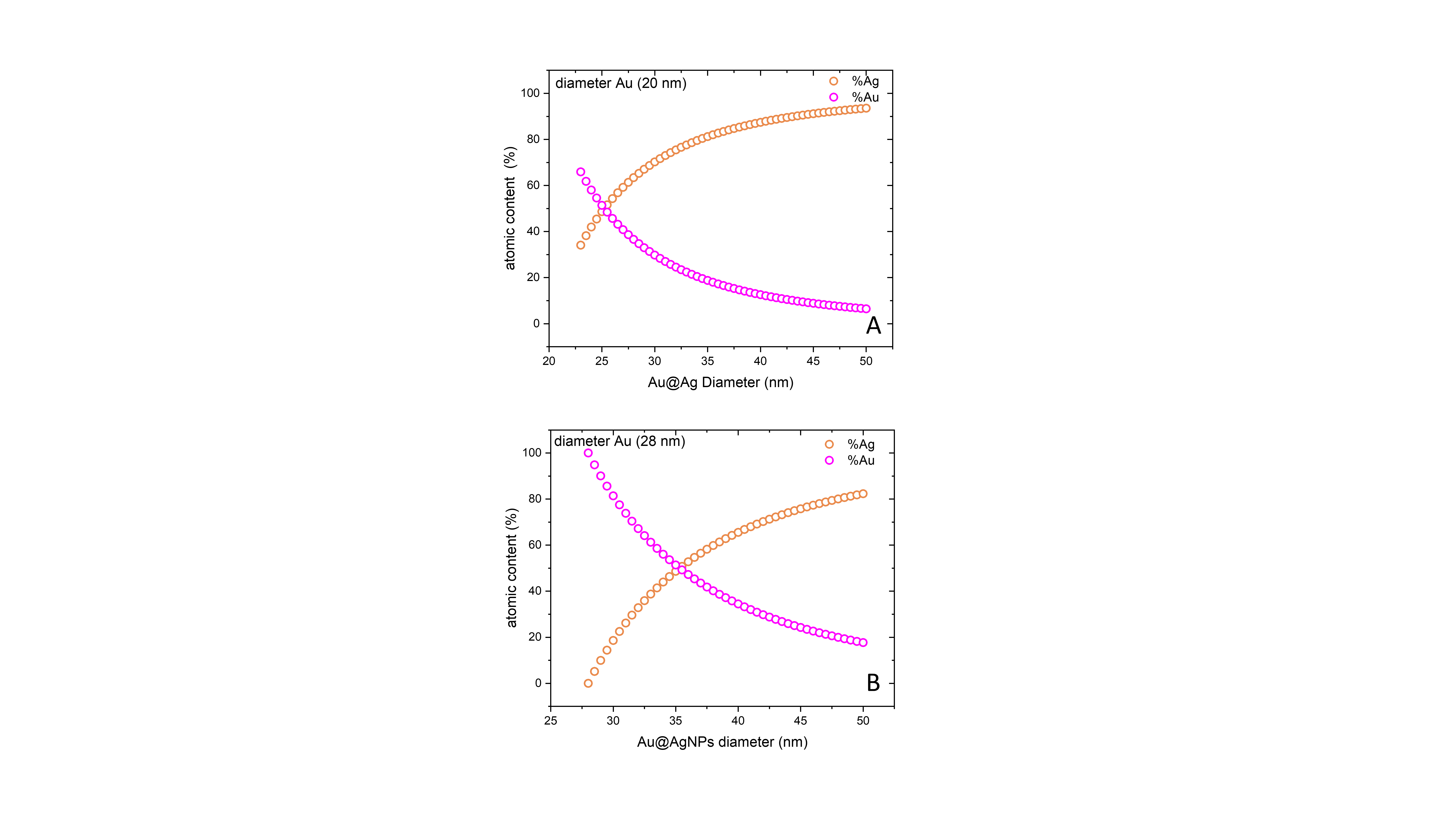
**

**Figure S7** Variations of atomic content percentage versus increase thickness shell.

**

**

**Figure S8** *C. albicans* dose-response curve comparative between Ag and Au@AgNPs.

Bacterial growth analysis using a modified Gompertz model and origin 2018 software. The parameters obtained of adjust are a, x_c_ y k, which are related to the following equations:

$$\mu=\frac{ka}{e}$$

Growth Rate µ

$$\lambda=\frac{k*x_{c}-1}{a}$$

and Time LagPhase $\lambda$.

***S.aureus* + AuNPs**

***S.aureus* without AuNPs**

**Table S1.** Adjust parameters of the curve by modified Gompertz Model (Origin 2018 software)

| **Parameter** | Mean Value | Standard Deviation |
| --- | --- | --- |
| **a** | 1.15528 | 0.00734 |
| **Xc** | 289.0 | 0.50 |
| **k** | 0.0090 | 1.3E-4 |
| R^2 ADJUST | 0.9801 |  |

**Table S2.** Physical Parameters of Bacterial Growth obtained with Adjust Parameters

| **Parameter** | Mean Value | Standard Deviation |
| --- | --- | --- |
| Growth Rate µ ($D.O./minutes)$ | $0.00383$ | $7.95526E-5$ |
| Time LagPhase $\lambda$ (*minutes)* | 177.8888 | $5.4467$ |

**Table S3.** ***S.aureus* + 1 µg/mL AuNPs**

Adjust parameters of the curve by modified Gompertz Model (Origin 2018 software)

| **PARAMETER** | MEAN VALUE | STANDARD DEVIATION |
| --- | --- | --- |
| **a** | 0.98751 | 0.00431 |
| **Xc** | 291.599 | 3.17369 |
| **k** | 0.0075 | 2.05E-4 |
| R^2 ADJUST | 0.99153 |  |

**Table S4.** PHYSICAL PARAMETERS OF BACTERIAL GROWTH OBTAINED WITH ADJUST PARAMETERS

| PARAMETER | Mean Value | Standard Deviation |
| --- | --- | --- |
| Growth Rate µ ($D.O./minutos)$ | $0.00272$ | $8.44162E-5$ |
| Time LagPhase $\lambda$ (*minutes)* | 158.2366 | $10.16166$ |

***S.aureus* + 10 µg/mL AuNPs**

**Table S5.** Adjust parameters of the curve by modified Gompertz Model (Origin 2018 software)

| **PARAMETER** | MEAN VALUE | STANDARD DEVIATION |
| --- | --- | --- |
| **a** | 0.91793 | 0.00471 |
| **Xc** | 315.5396 | 2.72545 |
| **k** | 0.0068 | 1.01252E-4 |
| R^2 ADJUST | 0..98746 |  |

**Table S6.** PHYSICAL PARAMETERS OF BACTERIAL GROWTH OBTAINED WITH ADJUST PARAMETERS

| PARAMETER | Mean Value | Standard Deviation |
| --- | --- | --- |
| Growth Rate µ ($D.O./minutos)$ | $0.0023$ | $4.5974E-5$ |
| Time LagPhase $\lambda$ (*minutes)* | 168.48078 | $6.4726$ |

***S.aureus* + 50 µg/mL AuNPs**

**Table S7.** Adjust parameters of the curve by modified Gompertz Model (Origin 2018 software)

| **PARAMETER** | MEAN VALUE | STANDARD DEVIATION |
| --- | --- | --- |
| **a** | 0.51 | 0.012 |
| **Xc** | 787.111 | 11.825 |
| **k** | 0.00615 | 3.989E-4 |
| R^2 ADJUST | 0..8844 |  |

**Table S8.** PHYSICAL PARAMETERS OF BACTERIAL GROWTH OBTAINED WITH ADJUST PARAMETERS

| PARAMETER | Mean Value | Standard Deviation |
| --- | --- | --- |
| Growth Rate µ ($D.O./minutos)$ | $0.00115$ | $1.01991E-4$ |
| Time LagPhase $\lambda$ (*minutes)* | 624.50937 | $90.39578$ |

***S.aureus* + AgNPs**

**Table S9.** ***S.aureus* without AgNPs**

Adjust parameters of the curve by modified Gompertz Model (Origin 2018 software)

| **PARAMETER** | MEAN VALUE | STANDARD DEVIATION |
| --- | --- | --- |
| **a** | 1.1122 | 0.00304 |
| **Xc** | 274.6266 | 1.69846 |
| **k** | 0.00904 | 1.77493E-4 |
| R^2 ADJUST | 0.9971 |  |

**Table S10.** PHYSICAL PARAMETERS OF BACTERIAL GROWTH OBTAINED WITH ADJUST PARAMETERS

| PARAMETER | Mean Value | Standard Deviation |
| --- | --- | --- |
| Growth Rate µ ($D.O./minutos)$ | $0.0037$ | $8.27335E-5$ |
| Time LagPhase $\lambda$ (*minutes)* | 164.00716 | $7.45461$ |

***S.aureus* 1µg/mL AgNPs**

**Table S11.** Adjust parameters of the curve by modified Gompertz Model (Origin 2018 software)

| **PARAMETER** | MEAN VALUE | STANDARD DEVIATION |
| --- | --- | --- |
| **a** | 0.90218 | 0.00282 |
| **Xc** | 269.47342 | 0.88062 |
| **k** | 0.01016 | 1.0549E-4 |
| R^2 ADJUST | 0.99931 |  |

**Table S12.** PHYSICAL PARAMETERS OF BACTERIAL GROWTH OBTAINED WITH ADJUST PARAMETERS

| PARAMETER | Mean Value | Standard Deviation |
| --- | --- | --- |
| Growth Rate µ ($D.O./minutes)$ | $0.00337$ | $4.55516E-5$ |
| Time LagPhase $\lambda$ (*minutes)* | 171.04822 | $4.11092$ |

***S.aureus* 10µg/mL AgNPs**

**Table S13.** Adjust parameters of the curve by modified Gompertz Model (Origin 2018 software)

| **PARAMETER** | MEAN VALUE | STANDARD DEVIATION |
| --- | --- | --- |
| **a** | 0.79089 | 0.01121 |
| **Xc** | 224.28364 | 2.04417 |
| **k** | 0.01616 | 6.61164E-4 |
| R^2 ADJUST | 0.98434 |  |

**Table S14.** PHYSICAL PARAMETERS OF BACTERIAL GROWTH OBTAINED WITH ADJUST PARAMETERS

| PARAMETER | Mean Value | Standard Deviation |
| --- | --- | --- |
| Growth Rate µ ($D.O./minutes)$ | $0.0047$ | $2.5901E-4$ |
| Time LagPhase $\lambda$ (*minutes)* | 162.40245 | $14.76911$ |

***S.aureus* 50µg/mL AgNPs**

**Table S15.** Adjust parameters of the curve by modified Gompertz Model (Origin 2018 software)

| **PARAMETER** | MEAN VALUE | STANDARD DEVIATION |
| --- | --- | --- |
| **a** | 0.74 | 0.001 |
| **Xc** | 967.54471 | 6.30715 |
| **k** | 0.00587 | 3.49063E-4 |
| R^2 ADJUST | 0.973 |  |

**Table S16.** PHYSICAL PARAMETERS OF BACTERIAL GROWTH OBTAINED WITH ADJUST PARAMETERS

| PARAMETER | Mean Value | Standard Deviation |
| --- | --- | --- |
| Growth Rate µ ($D.O./minutes)$ | $0.0016$ | $9.71851E-5$ |
| Time LagPhase $\lambda$ (*minutes)* | 797.18696 | $100.00702$ |

***S.aureus* + Au@AgNPs**

***S.aureus* without Au@AgNPs**

**Table S17.** Adjust parameters of the curve by modified Gompertz Model (Origin 2018 software)

| **PARAMETER** | MEAN VALUE | STANDARD DEVIATION |
| --- | --- | --- |
| **a** | 1.1122 | 0.00304 |
| **Xc** | 274.6266 | 1.69846 |
| **k** | 0.00904 | 1.77493E-4 |
| R^2 ADJUST | 0.9971 |  |

**Table S18.** PHYSICAL PARAMETERS OF BACTERIAL GROWTH OBTAINED WITH ADJUST PARAMETERS

| PARAMETER | Mean Value | Standard Deviation |
| --- | --- | --- |
| Growth Rate µ ($D.O./minutes)$ | $0.0037$ | $8.27335E-5$ |
| Time LagPhase $\lambda$ (*minutes)* | 164.00716 | $7.45461$ |

**S.*aureus* 1µg/mL Au@AgNPs**

**Table S19.** Adjust parameters of the curve by modified Gompertz Model (Origin 2018 software)

| **PARAMETER** | MEAN VALUE | STANDARD DEVIATION |
| --- | --- | --- |
| **a** | 0.90551 | 9.2098E-4 |
| **Xc** | 268.4542 | 1.16359 |
| **k** | 0.00926 | 1.20901E-4 |
| R^2 ADJUST | 0.99959 |  |

**Table S20.** PHYSICAL PARAMETERS OF BACTERIAL GROWTH OBTAINED WITH ADJUST PARAMETERS

| PARAMETER | Mean Value | Standard Deviation |
| --- | --- | --- |
| Growth Rate µ ($D.O./minutes)$ | 0.00308 | 4.34117E-5 |
| Time LagPhase $\lambda$ (*minutes)* | 160.46284 | 4.8856 |

***S.aureus* 10µg/mL Au@AgNPs**

**Table S21.** Adjust parameters of the curve by modified Gompertz Model (Origin 2018 software)

| **PARAMETER** | MEAN VALUE | STANDARD DEVIATION |
| --- | --- | --- |
| **a** | 0.76728 | 0.00446 |
| **Xc** | 283.117 | 2.55475 |
| **k** | 0.00968 | 1.9955E-4 |
| R^2 ADJUST | 0.99754 |  |

**Table S22.** PHYSICAL PARAMETERS OF BACTERIAL GROWTH OBTAINED WITH ADJUST PARAMETERS

| PARAMETER | Mean Value | Standard Deviation |
| --- | --- | --- |
| Growth Rate µ ($D.O./minutes)$ | 0.00273 | 7.22087E-5 |
| Time LagPhase $\lambda$ (*minutes)* | 179.81121 | 9.03605 |

***S.aureus* 50µg/mL Au@AgNPs**

**Table S23.** Adjust parameters of the curve by modified Gompertz Model (Origin 2018 software)

| **PARAMETER** | MEAN VALUE | STANDARD DEVIATION |
| --- | --- | --- |
| **a** | 0.73517 | 0.01493 |
| **Xc** | 802.288 | 14.5 |
| **k** | 0.00797 | 0.00104 |
| R^2 ADJUST | 0.99428 |  |

**Table S24.** PHYSICAL PARAMETERS OF BACTERIAL GROWTH OBTAINED WITH ADJUST PARAMETERS

| PARAMETER | Mean Value | Standard Deviation |
| --- | --- | --- |
| Growth Rate µ ($D.O./minutes)$ | 0.00216 | 3.25047E-4 |
| Time LagPhase $\lambda$ (*minutes)* | 676.81749 | 188.86726 |

***E.coli* + AuNPs**

***E.coli* without AuNPs**

**Table S25.** Adjust parameters of the curve by modified Gompertz Model (Origin 2018 software)

| **PARAMETER** | MEAN VALUE | STANDARD DEVIATION |
| --- | --- | --- |
| **a** | 0.83539 | .00546 |
| **Xc** | 284.94644 | 4.2278 |
| **k** | 0.00651 | 2.43998E-4 |
| R^2 ADJUST | 0.98643 |  |

**Table S26.** PHYSICAL PARAMETERS OF BACTERIAL GROWTH OBTAINED WITH ADJUST PARAMETERS

| PARAMETER | Mean Value | Standard Deviation |
| --- | --- | --- |
| Growth Rate µ ($D.O./minutes)$ | 0.002 | 8.80623E-5 |
| Time LagPhase $\lambda$ (*minutes)* | 131.33661 | 11.79379 |

***E.coli* + 1µg/mL AuNPs**

**Table S27.** Adjust parameters of the curve by modified Gompertz Model (Origin 2018 software)

| **PARAMETER** | MEAN VALUE | STANDARD DEVIATION |
| --- | --- | --- |
| **a** | .75065 | .00531 |
| **Xc** | 275.93547 | 4.77869 |
| **k** | 0.00666 | 2.79881E-4 |
| R^2 ADJUST | 0.98267 |  |

**Table S28.** PHYSICAL PARAMETERS OF BACTERIAL GROWTH OBTAINED WITH ADJUST PARAMETERS

| PARAMETER | Mean Value | Standard Deviation |
| --- | --- | --- |
| Growth Rate µ ($D.O./minutes)$ | 0.00184 | 9.02987E-5 |
| Time LagPhase $\lambda$ (*minutes)* | 125.78532 | 12.75042 |

***E.coli* + 10µg/mL AuNPs**

**Table S29.** Adjust parameters of the curve by modified Gompertz Model (Origin 2018 software)

| **PARAMETER** | MEAN VALUE | STANDARD DEVIATION |
| --- | --- | --- |
| **a** | 0.76901 | 0.00527 |
| **Xc** | 267.52651 | 4.67208 |
| **k** | 0.00658 | 2.67281E-4 |
| R^2 ADJUST | 0.98318 |  |

**Table S30.** PHYSICAL PARAMETERS OF BACTERIAL GROWTH OBTAINED WITH ADJUST PARAMETERS

| PARAMETER | Mean Value | Standard Deviation |
| --- | --- | --- |
| Growth Rate µ ($D.O./minutes)$ | 0.00186 | 8.83714E-5 |
| Time LagPhase $\lambda$ (*minutes)* | 115.55083 | 11.40538 |

***E.coli* + 50µg/mL AuNPs**

**Table S31.** Adjust parameters of the curve by modified Gompertz Model (Origin 2018 software)

| **PARAMETER** | MEAN VALUE | STANDARD DEVIATION |
| --- | --- | --- |
| **a** | 0.76171 | 0.00367 |
| **Xc** | 304.8801 | 3.18269 |
| **k** | 0.00644 | 1.77262E-4 |
| R^2 ADJUST | 0.99301 |  |

**Table S32.** PHYSICAL PARAMETERS OF BACTERIAL GROWTH OBTAINED WITH ADJUST PARAMETERS

| PARAMETER | Mean Value | Standard Deviation |
| --- | --- | --- |
| Growth Rate µ ($D.O./minutes)$ | 0.0018 | 5.83667E-5 |
| Time LagPhase $\lambda$ (*minutes)* | 149.6006 | 9.79726 |

***E.coli* + 100µg/mL AuNPs**

**Table S33.** Adjust parameters of the curve by modified Gompertz Model (Origin 2018 software)

| **PARAMETER** | MEAN VALUE | STANDARD DEVIATION |
| --- | --- | --- |
| **a** | 0.89091 | 0.00462 |
| **Xc** | 299.24812 | 3.447 |
| **k** | 0.00496 | 1.21086E-4 |
| R^2 ADJUST | 0.99318 |  |

**Table S34.** PHYSICAL PARAMETERS OF BACTERIAL GROWTH OBTAINED WITH ADJUST PARAMETERS

| PARAMETER | Mean Value | Standard Deviation |
| --- | --- | --- |
| Growth Rate µ ($D.O./minutes)$ | 0.00163 | 4.81157E-5 |
| Time LagPhase $\lambda$ (*minutes)* | 97.63522 | 5.89169 |

***E.coli* + AgNPs**

***E.coli* without AgNPs**

**Table S35.** Adjust parameters of the curve by modified Gompertz Model (Origin 2018 software)

| **PARAMETER** | MEAN VALUE | STANDARD DEVIATION |
| --- | --- | --- |
| **a** | 1.11606 | 0.00401 |
| **Xc** | 260.85241 | 2.32122 |
| **k** | 0.00795 | 1.90302E-4 |
| R^2 ADJUST | 0.99519 |  |

**Table S36.** PHYSICAL PARAMETERS OF BACTERIAL GROWTH OBTAINED WITH ADJUST PARAMETERS

| PARAMETER | Mean Value | Standard Deviation |
| --- | --- | --- |
| Growth Rate µ ($D.O./minutes)$ | 0.00326 | 8.98612E-5 |
| Time LagPhase $\lambda$ (*minutes)* | 135.06625 | 7.66816 |

***E.coli* + 1µg/mL AgNPs**

**Table S37.** Adjust parameters of the curve by modified Gompertz Model (Origin 2018 software)

| **PARAMETER** | MEAN VALUE | STANDARD DEVIATION |
| --- | --- | --- |
| **a** | 0.8839 | 0.00112 |
| **Xc** | 251.07818 | 0.84028 |
| **k** | 0.00844 | 7.54709E-5 |
| R^2 ADJUST | 0.99932 |  |

**Table S38.** PHYSICAL PARAMETERS OF BACTERIAL GROWTH OBTAINED WITH ADJUST PARAMETERS

| PARAMETER | Mean Value | Standard Deviation |
| --- | --- | --- |
| Growth Rate µ ($D.O./minutes)$ | 0.00274 | 2.80183E-5 |
| Time LagPhase $\lambda$ (*minutes)* | 132.59477 | 2.81509 |

***E.coli* + 10µg/mL AgNPs**

**Table S39.** Adjust parameters of the curve by modified Gompertz Model (Origin 2018 software)

| **PARAMETER** | MEAN VALUE | STANDARD DEVIATION |
| --- | --- | --- |
| **a** | 0.88798 | 0.00202 |
| **Xc** | 331.74964 | 1.4728 |
| **k** | .00579 | 6.8597E-5 |
| R^2 ADJUST | 0.99873 |  |

**Table S40.** PHYSICAL PARAMETERS OF BACTERIAL GROWTH OBTAINED WITH ADJUST PARAMETERS

| PARAMETER | Mean Value | Standard Deviation |
| --- | --- | --- |
| Growth Rate µ ($D.O./minutes)$ | 0.00189 | 2.67112E-5 |
| Time LagPhase $\lambda$ (*minutes)* | 159.03807 | 4.47445 |

***E.coli* + 50µg/mL AgNPs**

**Table S41.** Adjust parameters of the curve by modified Gompertz Model (Origin 2018 software)

| **PARAMETER** | MEAN VALUE | STANDARD DEVIATION |
| --- | --- | --- |
| **a** | 0.24357 | 0.00853 |
| **Xc** | 999.37678 | 8.83407 |
| **k** | 0.00703 | 6.84646E-4 |
| R^2 ADJUST | 0.96459 |  |

**Table S42.** PHYSICAL PARAMETERS OF BACTERIAL GROWTH OBTAINED WITH ADJUST PARAMETERS

| PARAMETER | Mean Value | Standard Deviation |
| --- | --- | --- |
| Growth Rate µ ($D.O./minutes)$ | 6.29919E-4 | 8.34075E-5 |
| Time LagPhase $\lambda$ (*minutes)* | 857.12927 | 174.52691 |

***E.coli* + 100µg/mL AgNPs**

**Table S43.** Adjust parameters of the curve by modified Gompertz Model (Origin 2018 software)

| **PARAMETER** | MEAN VALUE | STANDARD DEVIATION |
| --- | --- | --- |
| **a** | 0.16375 | 0.02129 |
| **Xc** | 1263.27391 | 14.211 |
| **k** | 0.01491 | 7.126E-4 |
| R^2 ADJUST | 0.64714 |  |

**Table S44.** PHYSICAL PARAMETERS OF BACTERIAL GROWTH OBTAINED WITH ADJUST PARAMETERS

| PARAMETER | Mean Value | Standard Deviation |
| --- | --- | --- |
| Growth Rate µ ($D.O./minutos)$ | 8.98182E-4 | 1.59705E-4 |
| Time LagPhase $\lambda$ (*minutes)* | 1196.20483 | 127.79797 |

***E.coli* + Au@AgNPs**

***E.coli* without Au@AgNPs**

**Table S45.** Adjust parameters of the curve by modified Gompertz Model (Origin 2018 software)

| **PARAMETER** | MEAN VALUE | STANDARD DEVIATION |
| --- | --- | --- |
| **a** | 1.11762 | .00371 |
| **Xc** | 267.82612 | 2.214 |
| **k** | 0.0077 | 1.68698E-4 |
| R^2 ADJUST | 0.99587 |  |

**Table S46.** PHYSICAL PARAMETERS OF BACTERIAL GROWTH OBTAINED WITH ADJUST PARAMETERS

| PARAMETER | Mean Value | Standard Deviation |
| --- | --- | --- |
| Growth Rate µ ($D.O./minutes)$ | 0.00317 | 7.98693E-5 |
| Time LagPhase $\lambda$ (*minutes)* | 137.95599 | 7.18533 |

***E.coli* + 1µg/mL Au@AgNPs**

**Table S47.** Adjust parameters of the curve by modified Gompertz Model (Origin 2018 software)

| **PARAMETER** | MEAN VALUE | STANDARD DEVIATION |
| --- | --- | --- |
| **a** | 0.90492 | .00157 |
| **Xc** | 258.17945 | 1.15103 |
| **k** | 0.00812 | 9.64081E-5 |
| R^2 ADJUST | 0.99879 |  |

**Table S48.** PHYSICAL PARAMETERS OF BACTERIAL GROWTH OBTAINED WITH ADJUST PARAMETERS

| PARAMETER | Mean Value | Standard Deviation |
| --- | --- | --- |
| Growth Rate µ ($D.O./minutes)$ | 0.0027 | 3.67843E-5 |
| Time LagPhase $\lambda$ (*minutes)* | 135.02674 | 3.80831 |

***E.coli* + 10µg/mL Au@AgNPs**

**Table S49.** Adjust parameters of the curve by modified Gompertz Model (Origin 2018 software)

| **PARAMETER** | MEAN VALUE | STANDARD DEVIATION |
| --- | --- | --- |
| **a** | 0.88673 | 0.00207 |
| **Xc** | 432.0806 | 1.3607 |
| **k** | 0.00578 | 6.48403E-5 |
| R^2 ADJUST | 0.99907 |  |

**Table S50.** PHYSICAL PARAMETERS OF BACTERIAL GROWTH OBTAINED WITH ADJUST PARAMETERS

| PARAMETER | Mean Value | Standard Deviation |
| --- | --- | --- |
| Growth Rate µ ($D.O./minutes)$ | 0.00189 | 2.55531E-5 |
| Time LagPhase $\lambda$ (*minutes)* | 259.07022 | 6.62838 |

***E.coli* + 50µg/mL Au@AgNPs**

**Table S51.** Adjust parameters of the curve by modified Gompertz Model (Origin 2018 software)

| **PARAMETER** | MEAN VALUE | STANDARD DEVIATION |
| --- | --- | --- |
| **a** | 0.48624 | .02697 |
| **Xc** | 1355.71203 | 4.580 |
| **k** | 0.01526 | 9.04012E-4 |
| R^2 ADJUST | 0.98815 |  |

**Table S52.** PHYSICAL PARAMETERS OF BACTERIAL GROWTH OBTAINED WITH ADJUST PARAMETERS

| PARAMETER | Mean Value | Standard Deviation |
| --- | --- | --- |
| Growth Rate µ ($D.O./minutos)$ | 0.00273 | 3.13113E-4 |
| Time LagPhase $\lambda$ (*minutes)* | 1290.18123 | 157.22091 |

***E.coli* + 100µg/mL Au@AgNPs**

**Table S53.** Adjust parameters of the curve by modified Gompertz Model (Origin 2018 software)

| **PARAMETER** | MEAN VALUE | STANDARD DEVIATION |
| --- | --- | --- |
| **a** | 0.03487 | 3.31568E-4 |
| **Xc** | 1721.68697 | 5.0138 |
| **k** | 0.00351 | 2.81594E-5 |
| R^2 ADJUST | 0.99414 |  |

**Table S54.** PHYSICAL PARAMETERS OF BACTERIAL GROWTH OBTAINED WITH ADJUST PARAMETERS

| PARAMETER | Mean Value | Standard Deviation |
| --- | --- | --- |
| Growth Rate µ ($D.O./minutes)$ | 4.50261E-5 | 7.89367E-7 |
| Time LagPhase $\lambda$ (*minutes)* | 1436.78669 | 27.23772 |


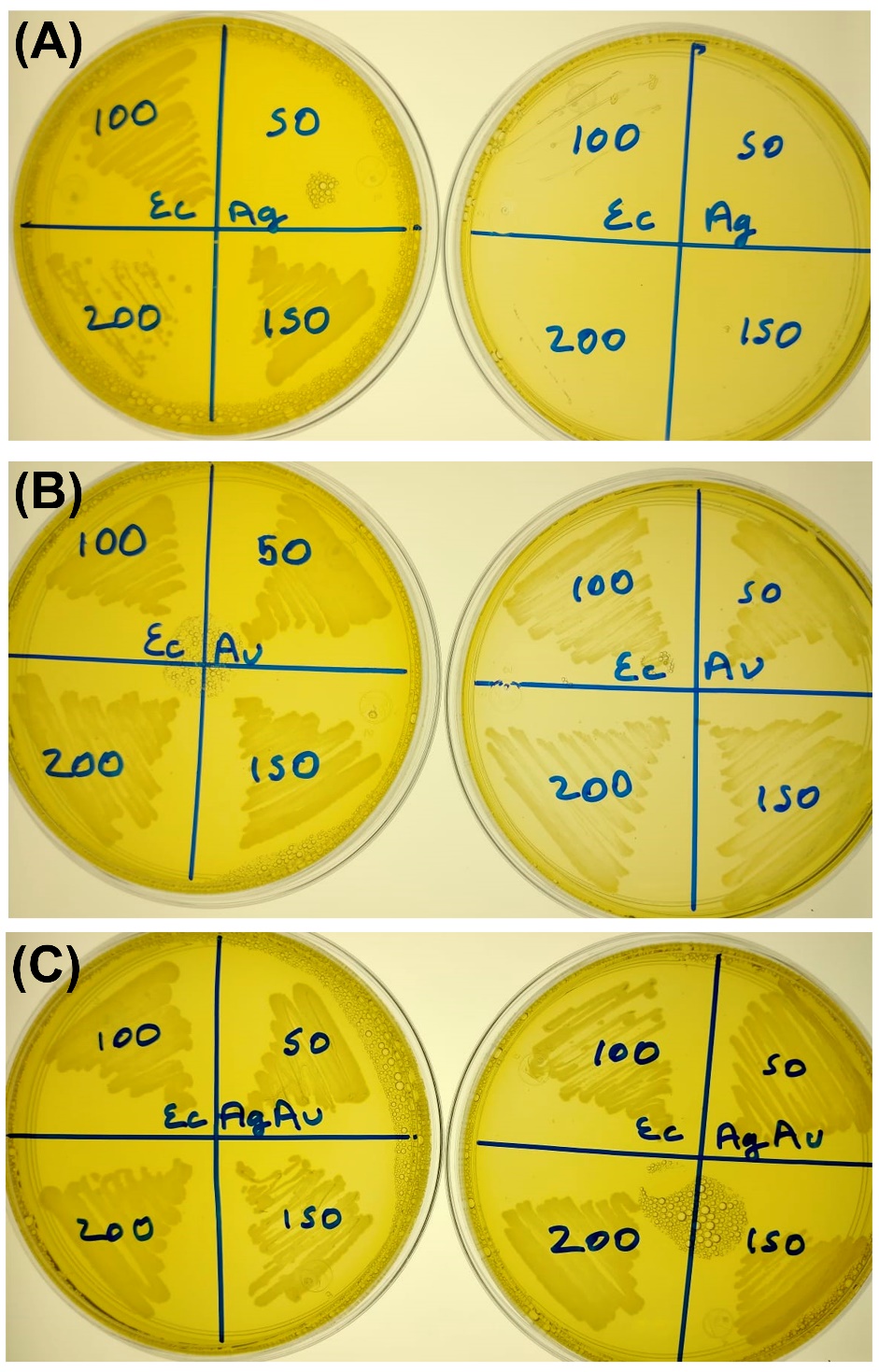


**Figure S9.** Minimal Bactericidal Concentration determined for *E. coli* treated with nanoparticles and inoculated in Muller Hinton plate. AgNPs (A), AuNPs (B), and Au@AgNPs (C)


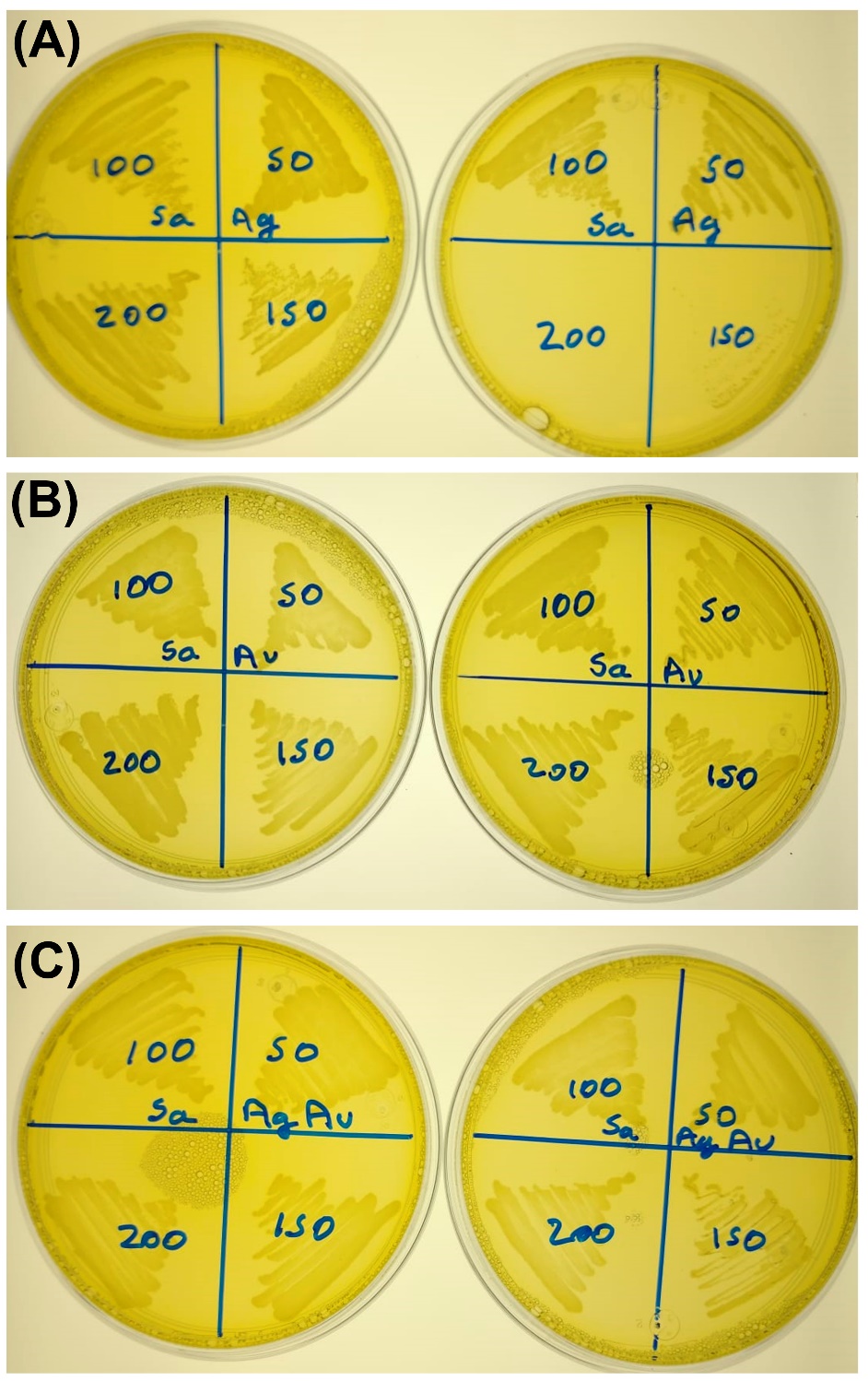


**Figure S10.** Minimal Bactericidal Concentration determined for *S. aureus* treated with nanoparticles and inoculated in Muller Hinton plate. AgNPs (A), AuNPs (B), and Au@AgNPs (C)


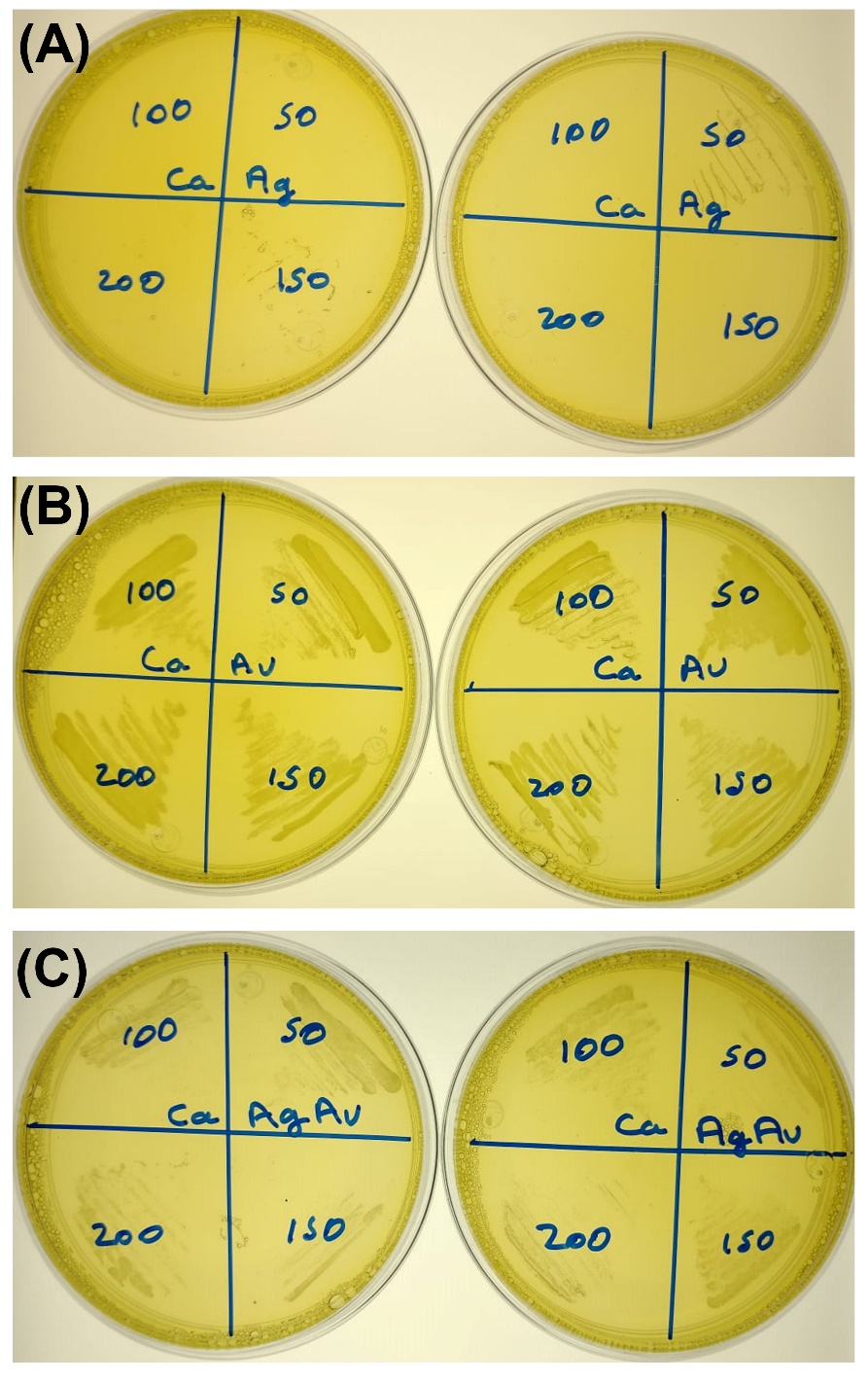


**Figure S11.** Minimal Bactericidal Concentration determined for *C. albicans* treated with nanoparticles and inoculated in Muller Hinton plate. AgNPs (A), AuNPs (B), and Au@AgNPs (C)

**Table S55.** Minimal Bactericidal concentration (MBC).

| *Escherichia coli* ATCC 25922 | | | | | |
| --- | --- | --- | --- | --- | --- |
|  | 200 | 150 | 100 | 50 | 0 |
| AgNPs | + | + | + | + | + |
| AuNPs | + | + | + | + | + |
| Ag@AuNPs | + | + | + | + | + |
| *Staphylococcus aureus* ATCC 6538P | | | | | |
|  | 200 | 150 | 100 | 50 | 0 |
| AgNPs | + | + | + | + | + |
| AuNPs | + | + | + | + | + |
| Ag@AuNPs | + | + | + | + | + |
| *Candida albicans* | | | | | |
|  | 200 | 150 | 100 | 50 | 0 |
| AgNPs | - | - | - | - | + |
| AuNPs | + | + | + | + | + |
| Ag@AuNPs | + | + | + | + | + |
